# Sequestration of Small Ions and Weak Acids and Bases by a Polyelectrolyte Complex Studied by Simulation and Experiment

**DOI:** 10.1021/acs.macromol.3c01209

**Published:** 2024-01-18

**Authors:** Roman Staňo, Jéré J. van Lente, Saskia Lindhoud, Peter Košovan

**Affiliations:** † Faculty of Physics, University of Vienna, Boltzmanngasse 5, 1090 Vienna, Austria; ‡ Vienna Doctoral School in Physics, University of Vienna, Boltzmanngasse 5, 1090 Vienna, Austria; § Department of Molecules & Materials, University of Twente, Drienerlolaan 5, 7522 NB Enschede, The Netherlands; ∥ Department of Physical and Macromolecular Chemistry, Faculty of Science, Charles University, Hlavova 8, 128 40 Prague 2, Czech Republic

## Abstract

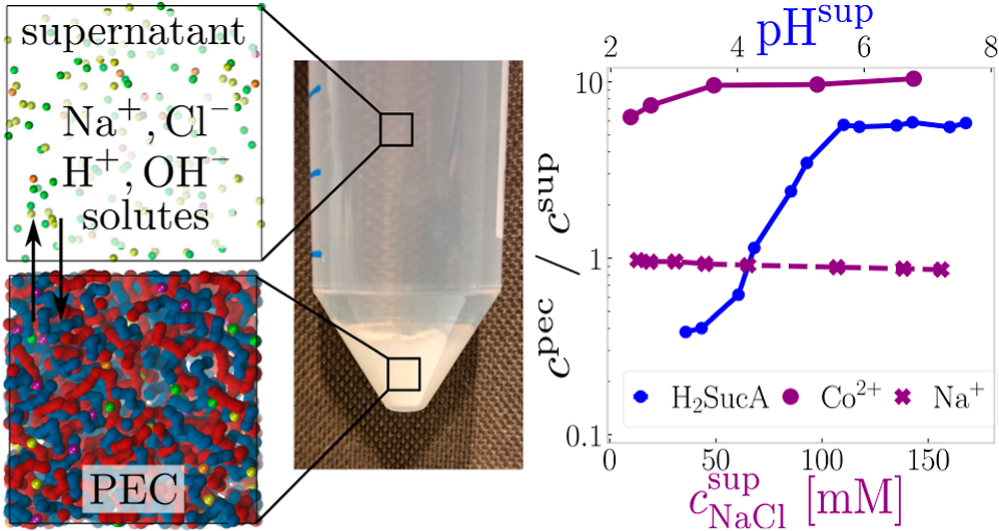

Mixing of oppositely charged polyelectrolytes can result in phase separation into a polymer-poor supernatant and a polymer-rich polyelectrolyte complex (PEC). We present a new coarse-grained model for the Grand-reaction method that enables us to determine the composition of the coexisting phases in a broad range of pH and salt concentrations. We validate the model by comparing it to recent simulations and experimental studies, as well as our own experiments on poly(acrylic acid)/poly(allylamine hydrochloride) complexes. The simulations using our model predict that monovalent ions partition approximately equally between both phases, whereas divalent ones accumulate in the PEC phase. On a semiquantitative level, these results agree with our own experiments, as well as with other experiments and simulations in the literature. In the sequel, we use the model to study the partitioning of a weak diprotic acid at various pH values of the supernatant. Our results show that the ionization of the acid is enhanced in the PEC phase, resulting in its preferential accumulation in this phase, which monotonically increases with the pH. Currently, this effect is still waiting to be confirmed experimentally. We explore how the model parameters (particle size, charge density, permittivity, and solvent quality) affect the measured partition coefficients, showing that fine-tuning of these parameters can make the agreement with the experiments almost quantitative. Nevertheless, our results show that charge regulation in multivalent solutes can potentially be exploited in engineering the partitioning of charged molecules in PEC-based systems at various pH values.

## 1 Introduction

Mixing of oppositely charged macromolecules can result in the formation of polyelectrolyte complexes (PECs).^1−4^ This associative process is often accompanied by a phase separation, in which the system demixes into two phases: the polymer-rich PEC phase, coexisting with a dilute supernatant phase which is almost free of the polymers. When the PEC phase is liquid, it is termed a complex coacervate.^5,6^ Complex coacervates have found use in hygiene products,^7^ food industry,^8^ and they are prospective candidates for medical applications,^9−12^ water purification,^13−15^ or development of programmable materials.^16^ Furthermore, the mechanism of coacervation inspired a plethora of soft materials based on the assembly of polyelectrolytes with other charged objects, such as multivalent macroions,^17,18^ colloids,^19^ and copolymers yielding coacervate core micelles^20,21^ or proteins.^22−24^ Naturally, the use of PECs in such delicate arrangements requires a high level of fundamental understanding of the underlying physics and chemistry that govern the properties of these materials.

Recently, it has been recognized that cells utilize coacervation and liquid–liquid phase separation^25−28^ to compartmentalize and regulate their matter and to create microenvironments^29−31^ akin to bionanoreactors for catalyzing chemical reactions or protein folding. This recognition sparked new interest in the old phenomenon of complex coacervation. Furthermore, it has been demonstrated that coacervates made of synthetic polyelectrolytes can be used to sequestrate proteins,^32−39^ similar to the membraneless organelles which can selectively encapsulate proteins from cytoplasm. Such an uptake can be not only selective but also can preserve the activity^40^ and secondary structure of proteins,^41^ which is necessary for potential applications in drug delivery or peptide therapy.^33^ In addition, sequestration of proteins by coacervates can be regulated by change of pH or mixing ratio of polyelectrolytes.^40,42^ Generally, it seems to be clear that electrostatic interactions play a key role in the partitioning, whereas hydrophobicity or other specific interactions fine-tune the behavior while the liquid nature of both phases ensures that the whole system remains close to thermodynamic equilibrium.

Systematic exploration of thermodynamic stability of coacervates established phase diagrams as a function of chain length, mixing ratio, pH, or salt concentration.^43−49^ The general idea seems to be to use experimental setups with a minimal number of well-defined components, such that their properties can be varied systematically. Other studies, focusing for instance on the thermodynamics of the complex formation,^50,51^ viscoelasticity,^52−57^ or interfacial properties,^58,59^ show that the coacervate phase behavior is universal to a high degree. Concurrently, theoretical approaches,^60^ such as scaling (blob) arguments,^61,62^ mean-field and liquid-state theories,^63−70^ field-theoretic calculations^71^ or transfer-matrix formalism,^72−74^ have identified the key actors: charge correlations,^75^ counterion condensation,^76^ or chain connectivity.^77,78^ Additionally, it has been shown that specific chemistry of the polymer,^79−82^ counterions,^83,84^ or solvent^85^ can strongly affect the stability and composition of the complexes. Nevertheless, the agreement between the experiment and theory remained mostly qualitative. While the experimental results reflect on both generic physical and specific chemical effects, the theories can typically systematically describe only the former effect, whereas the latter often enters the calculations in the form of phenomenological material constants, which are hard to evaluate or predict from theory. To enable further progress, it seems necessary to disentangle the universal and system-specific effects and understand how they both contribute to the net result.

The partitioning of solutes between the coacervate and supernatant remains a challenge for theory and modeling. It is still poorly understood even for simple ions, let alone more complex organic molecules or proteins. In thermodynamic equilibrium, the chemical potential of each species *i*, which can be exchanged between the two phases (PEC and supernatant), must be the same
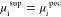
1

This chemical potential can be separated into the reference, ideal gas, and excess contributions
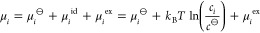
2

By combining eqs 1 and 2, the reference chemical potentials cancel, and we obtain an expression for the partition coefficient of species *i*, defined as the ratio of its concentrations in the supernatant and PEC phase
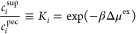
3

Clearly, this partition coefficient is controlled by the excess chemical potentials, and the main challenge for theory and simulations is to correctly predict this term. In general, the excess chemical potential includes steric, short-range (van der Waals), and electrostatic contributions, and a Donnan contribution. If the coacervate is charge-balanced, then the Donnan contribution is zero. In such a case, the partition coefficient is predominantly controlled by the subtle balance between the electrostatic interactions and steric effects, whereas the system-specific short-range interactions further modulate the result. None of these contributions can be negligible in a dense multicomponent system, such as the coacervate phase; therefore, their estimation remains the main theoretical challenge. For example, many theoretical models and simulations predict that monovalent ions should preferentially accumulate in the coacervate phase,^4^ whereas experiments show that simple salts, such as NaCl, slightly prefer the supernatant phase.^86^ It took several decades to establish a qualitative explanation of this observation.^67,72,84,86^

While many theories and experiments investigated the partitioning of monovalent salts, much less is known about the partitioning of multivalent ions^87,88^ or small organic molecules.^14,89−91^ In brief, these experimental studies have shown that multivalent ions generally tend to accumulate in the PEC phase. Furthermore, the partitioning of multivalent solutes depends also on their size, hydrophobicity, and charge. Thus, it is clear that the partitioning of organic and multivalent molecules is strongly affected by short-range interactions and charge–charge correlations, in addition to the effects that determine the partitioning of monovalent salt ions. Nevertheless, a systematic understanding of how these parameters affect the partitioning has not yet been fully established yet.

Considering the number of experiments utilizing a change of pH to regulate the coacervation, it seems surprising that it has not yet received much attention in molecular simulations. The term charge regulation refers to a change in the ionization states of weakly acidic or basic groups, depending on the pH and their local environment. Within the mean-field picture, the degree of ionization, α, can be described by an augmented Henderson–Hasselbalch equation^92^
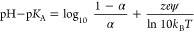
4where *ze* is the charge and ψ is the mean electrostatic potential. Thus, the partitioning of charge-regulating solutes is affected by the difference in ψ between coacervate and the supernatant in two different ways: (1) at fixed valency of the solute, it determines the electrostatic contribution to the excess chemical potential; (2) at pH which is not too far from p*K*_A_ of the solute, valency of the solute can be switched as it is exchanged between the phases. While the first effect has been considered in the existing theories, the second one has not yet been analyzed theoretically, although there is clear experimental evidence that a change in pH dramatically affects solute partitioning.^42^ Notably, a very recent study by Choi et al.^91^ has shown that a weakly acidic solute is more ionized in the PEC phase than that in the supernatant, indicating a complicated interplay between the acid–base equilibrium and solute partitioning.

To bridge this knowledge gap between theory and experiments, various molecular models have been designed for use in molecular simulations, which have become a strong tool for the polymer physics.^93^ The prevalent approaches in particle-based simulations of PECs can be classified into three categories. First, simulations exploring only a single pair of oppositely charged chains in dilute conditions:^94−96^ these studies correctly identified the main thermodynamic driving forces of polyelectrolyte complexation, but they provided very little information about the behavior of the complexes in the bulk. Second, simulations using many chains in a large simulation cell, directly emulating the phase separation:^97−100^ in these simulations, a nanodroplet, a slab, or an aggregate of PEC is formed inside an otherwise empty box. Such simulations suffer from massive finite-size effects caused by the explicit interface of the coacervate nanodroplet. Extrapolation of these results to the macroscopic systems is problematic not only because the interface contributes much less to the bulk properties but also because the bulk of the coacervate phase is poorly defined in such simulation. Third group of the simulations is based on the Gibbs ensemble,^86,101,102^ where one simulates coexistence of two bulk phases (PEC and supernatant) using two simulation cells, avoiding the need for simulating their interface.^103^ Such a simulation typically assumes that there is no polymer in the supernatant phase, while simultaneously, small ions and other solutes can be exchanged between both phases. This third group of simulations is well suited for predicting the phase stability of coacervates and the partitioning of solutes between the phases. It avoids the drawbacks of the previous two; therefore, the only limitation of this group consists in the quality of the molecular model. Indeed, the simulations of the third group qualitatively reproduced the experimentally observed phase diagrams as a function of salt concentration and also partitioned monovalent ions. In addition, by tuning the model parameters, a quantitative agreement between such simulations and specific experiments could be achieved.^49,86,101^

Our method falls within this third category; however, in contrast to the previous studies, it also includes the effects of pH and charge regulation. These effects have not yet been addressed in molecular simulations although they have been addressed by phenomenological thermodynamic models.^64,104,105^ Unlike the preceding simulation studies, we have not designed our model to reproduce the properties of a specific experimental system. Instead, our models of polymers and solutes are constructed as a generic bead–spring models, possessing only a small number of parameters which can be linked with solvent permittivity, size and valency of the monomers and of the small ions. Such a setting allows us to systematically explore the effect of these parameters on the phase stability of the PEC and on the partitioning of small ions. Only in simulations, these parameters can be varied independently while keeping all other interactions constant, thereby allowing us to distinguish generic features of the phase separation and partitioning from those which are determined by the specific chemistry of the polymers and small ions.

We use the Grand-reaction Monte Carlo method,^106,107^ which allows us to simulate coexistence between the supernatant and coacervate phases and chemical equilibrium within each phase in a broad range of salt concentrations, pH values, and in the presence of small molecular or ionic solutes. The Grand-reaction method guarantees the equality of chemical potentials of the partitioning species between the phases and the equality of osmotic pressures between the phases, and it also provides an option to assess its self-consistency. Similar to the previous studies, our model assumes that there is no polymer in the supernatant phase. This approximation works well far from the critical point, whereas it fails in the critical region, which defines the range of applicability of our approach. In the current work, we use only conventional trial Monte Carlo insertion moves, which can become inefficient when simulating the partitioning of bulky solutes. This deficiency can be suppressed by coupling our method to the continuous fractional component ansatz^108,109^ or to thermodynamic integration, which we plan to do in the future.

Although our model has not been fine-tuned to reproduce a specific experimental system, we first validate our simulation results against published experimental data on the partitioning of NaCl.^86^ Second, we describe the partitioning of CoCl_2_ by comparing our simulations with our experiments using complexes of poly(acrylic acid) (PAA) and poly(allylamine hydrochloride) (PAH), again reaching a nearly quantitative agreement. Next, we use the simulations to predict partitioning of diprotic succinic acid as a function of pH, observing a strong coupling between the acid–base equilibrium and partitioning. Finally, we systematically examine the sensitivity of our simulation results to parameters of the model, showing that fine-tuning of these parameters would allow us to reach a quantitative agreement with experiments.

## 2 Model and Method

### 2.1 Microscopic Model for the Simulations

**Figure 1:**
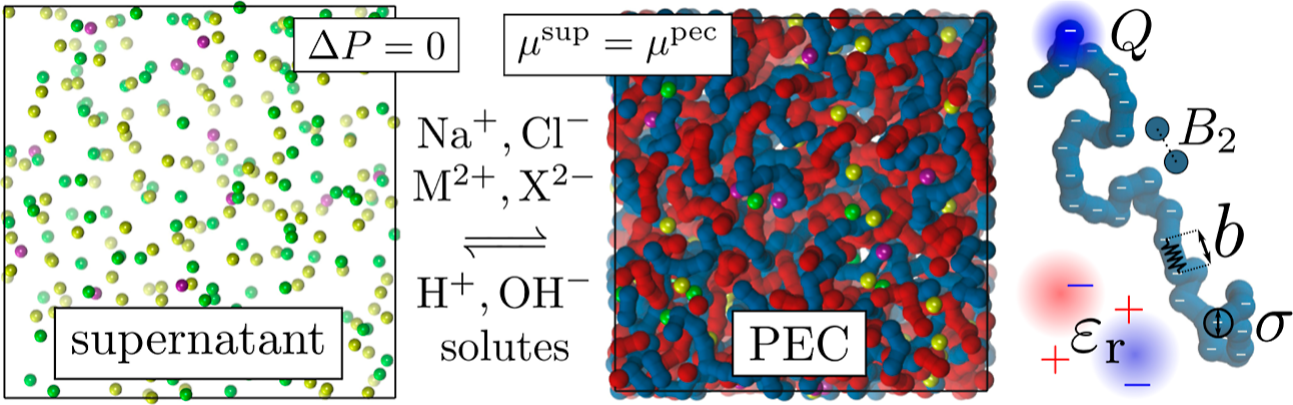
Simulation model: supernatant phase (left) indirectly coupled to the polyelectrolyte complex phase (PEC, right). The snapshot shows PEC in equilibrium with the supernatant at . Polyanion is blue; polycation is red; and Na^+^, Cl^–^, and M^2+^ ions are yellow, green, and purple, respectively. The simulation snapshot on the far right shows a representative conformation of a single chain in the PEC phase. Symbols in the snapshot represent the effective monomer size σ, its charge *Q*, the effective bond length *b*, the second virial coefficient *B*_2_, and the permittivity ε_r_.

To represent the PEC in equilibrium with the supernatant in silico, we opt for the approach used in our previous studies on multiphase polyelectrolyte systems.^106,110−112^ We independently simulate a concentrated solution of polyelectrolytes, representing the bulk of the PEC phase, and a salt solution, representing the bulk of the supernatant phase, as captured in Figure 1. The two phases are indirectly coupled, as detailed in the following subsections.

Our main objective is to devise a minimal physical model that can readily explain some experimental observations. By keeping the amount of variable parameters small we sacrifice chemical specificity for the sake of model simplicity. Therefore, we do not perform any systematic coarse-graining from the atomistic structure of PAA or PAH. Instead, we choose the parameters in a reasonable range, based on semiempirical estimates within the range used for similar models in the literature, roughly matching the key features of some common experimental systems. In this section, we only list the default values of model parameters. Later, in Section 3.4, we present a systematic analysis and a detailed discussion of how the choices of these parameters affect our numerical results, namely, monomer size, charge density on the polymer, permittivity of the PEC phase, solvent quality, and overall system size.

#### 2.1.1 Polyelectrolyte Complex Phase

The phase of the PEC, denoted by superscript pec, consists of *N*_A_ = 32 polyanions and *N*_C_ = 32 polycations of length *M* = 32 units each. The simulated system is enclosed in a cubic simulation box of size *L* with periodic boundary conditions. Later, in Section 3.4.3, we demonstrate that this system size proved to be sufficient to avoid significant finite-size effects. Monomeric units of the polyelectrolyte chains are modeled as spherical particles characterized by effective size, σ_mon_, and fixed charge number, *z*_mon_. Unless stated otherwise, σ_mon_ = 0.426 nm = 1.2σ_ion_ for both polycation and polyanion, and *z* = ± 1, respectively, where the choice of the effective size of ions, σ_ion_, is explained below. Each pair of monomeric units interacts through the nonbonded pairwise potential introduced by Weeks, Chandler, and Andersen (WCA)^113,114^
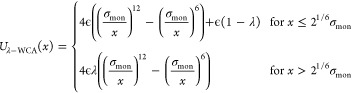
5where *x* is the instantaneous distance, ϵ = *k*_B_*T* = 1/β, and λ is the parameter controlling the hydrophobicity. Further details are provided in Section S1.1 of the Supporting Information, where we also plot the potential in Figure S1 and relate the values of λ to the second virial coefficient, *B*_2_.^115^ We always use the truncated and shifted form^116^ of the potential in eq 5 with the cutoff of 2.5σ_mon_ = 1.065 nm. Unless stated otherwise, we use λ = 0, yielding a purely repulsive WCA potential, which represents good (athermal) solvent conditions.

Next, to represent the polymer connectivity, we use harmonic bonds governed by the potential
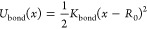
6where *K*_bond_ = 827 *k*_B_*T*/nm^2^ is the spring stiffness and *R*_0_ = σ_mon_ = 0.426 nm, unless stated otherwise. The combination of potentials yields the average bond length *b* ≈ 0.438 ± 0.025 nm, where the first value is the estimated mean and the second value is the estimated standard deviation.

Besides the polymer chains, the PEC phase also contains small ions and other small solutes. These include the H^+^, OH^–^ and salt ions Na^+^, Cl^–^. Additional solutes are present in some cases, such as generic divalent ions, denoted as M^2+^, X^2–^, or a small diprotic weak acid (succinic acid) in all of its ionization forms H_2_SuA, HSuA^–^, and SuA^2–^. All solutes are being exchanged with the supernatant, as described in Section 2.2, and hence their numbers fluctuate. They are all represented as spherical particles with λ = 0, an effective diameter σ_ion_ = 0.355 nm, and the respective charge number *z*_*i*_, as depicted in Figure 1 and listed in Table S2 in the Supporting Information. The above choice of ion diameter reasonably well represents the excess chemical potential of small ionic solutes in aqueous solutions, such NaCl, almost up to the solubility limit.^106^ Interactions between the solutes and monomeric units of the polymers are modeled by purely repulsive WCA potential, where we accordingly adjust the length scale of the potential to (σ_ion_ + σ_mon_)/2 using the Lorentz–Berthelot combination rule.^116^

In addition to the interactions described above, each pair of charged particles (small ions, other solutes, and monomeric units of the polymer) interacts via the Coulombic potential
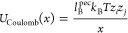
7where *l*_B_^pec^ = *e*^2^/4πε_0_ε_r_*k*_B_*T* = 0.89 nm is the Bjerrum length of the implicit solvent, which is represented as a dielectric continuum. This value corresponds to the relative permittivity of the PEC phase, ε_r_ ≈ 62. The electrostatic potential and forces are evaluated using the particle–particle particle–mesh (PPPM) method,^117^ where we require the relative error^118,119^ of the method to be ≤10^–3^.

#### 2.1.2 Supernatant Phase

The phase of the supernatant, denoted by the superscript sup, contains only small ions and other solutes in implicit solvent, but it does not contain any polymer. Unlike the PEC phase, we set the Bjerrum length in the supernatant to *l*_B_^sup^ = 0.71 nm, which corresponds to the permittivity of pure water at room temperature, ε_r_ ≈ 78. Otherwise, all other interactions in the supernatant phase are the same as those in the PEC phase.

#### 2.1.3 Validity of Some Employed Approximations

When constructing the model, we made some approximations that may not always be satisfied. These approximations define the range of applicability of our model and of the whole approach to modeling the phase-coexistence and the formation of PECs.

##### 2.1.3.1 Absence of the Polymer in the Supernatant Phase

We assume that the supernatant phase does not contain any polymer,^86^ and also our experiments in Section 3.2, show that this is indeed a reasonable assumption for chains as short as *M* = 50, provided that the two phase system is far enough from the critical point. Admittedly, because of this assumption, our method is unable to reconstruct the full phase diagrams, leaving the regions around the critical point unrendered. Nevertheless, as we show later, our method allows self-consistent assessment of its validity, and hence, the requirement of subcriticality can be tested.

##### 2.1.3.2 Fixed Charges on the Polymers

In the current simulations, we assume that the charges on the polymers are fixed, independent of pH, as if the polymers were strong polyelectrolytes. In contrast, the polymers used in our experiments, PAA and PAH, are weak polyelectrolytes, which can attain different degrees of charging, depending on pH. However, the experiments were carried out at pH ≈ 7, where the Henderson–Hasselbalch equation predicts that both ideal polycation and polyanion are fully charged and hence effectively quenched. Although, the real system is nonideal, both experiments on PECs^83,120^ and simulations of electrostatically cross-linked gels^112^ suggest that charge regulation of PECs should be close to the ideal one, as described by the Henderson–Hasselbalch equation. Arguably, the charge regulation can become a nontrivial actor, especially if the polyanion-polycation mixing ratio is not unity, due to the emerging Donnan potential between the phases. In principle, the Monte Carlo in the Grand-reaction ensemble (G-RxMC) method used here is able to quantitatively capture these effects, as repeatedly proven in simulations of gels and peptide solutions.^121−123^ Herein, we focus only on the charge regulation of a small solute in the presence of an PEC. The effects of charge regulation and pH on the formation of PECs will be addressed in detail in our follow-up publication.

##### 2.1.3.3 Solvation of Ions

In general, we assume different permittivities in the phase of supernatant and the complex. This would imply different interactions between the ions and solvent in the two phases, yielding a nonzero solvation contribution to the chemical potential of ions. Due to the coarse-grained nature of the model and use of implicit solvent, we cannot properly estimate this contribution, so we just neglect it. As a rough estimate of the associated systematic error for this approximation, we can invoke the Born equation, which gives us βμ^S^ = *z*^2^(*l*_B_^pec^ – *l*_B_^sup^)/2*r*_0_, where μ^S^ is the solvation contribution to the chemical potential, connected with a transfer of a single ion of size *r*_0_ and valency *z* from the supernatant to the complex phase. For the ionic pair NaCl, this contribution amounts to , possibly affecting the partition coefficients by multiplicative factor exp(0.5) ≈ 1.6, which is close to unity. Nevertheless, we underline that the Born equation is just an approximate continuum theory, and its validity inside the complex phase is questionable. Better understanding of the role of solvation of ions would require using atomistic simulations with explicit solvent, which goes beyond the scope of the current study.

### 2.2 Simulation Method

Our simulations comprise dynamic steps, sampling the conformations of the system, overlaid by reaction steps, sampling fluctuations in the composition of the system.^92^ For the former type of steps, we use Langevin dynamics in canonical ensemble, while for the latter, we use the G-RxMC method.^106,110^ All simulations were carried out using the Extensible Simulation Package for Research on Soft Matter (ESPResSo), release version 4.1.4.^124^

#### 2.2.1 Dynamic Steps—Langevin Dynamics

The governing equations of motion for Langevin dynamics for each of the particles are
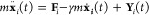
8where γ is the friction coefficient and **Y** is a random force obeying ⟨**Y**_*i*_^*p*^(*t*)**Y**_*j*_^*q*^(*t*^′^)⟩ = 2γ*m*_*i*_*k*_B_*T*δ_*ij*_*δ*_*pq*_δ(*t* – *t*^′^) and ⟨**Y**_*i*_^*p*^(*t*)⟩ = 0, where *p*, *q* ∈ {*x*, *y*, *z*} are Cartesian coordinates, δ is the Kronecker delta, and **F**_*i*_ is the deterministic force acting on the particle, originating from the gradient of potentials, given by eqs 5–7. We set the mass of each particle to *m* = 1, which in conjunction with σ = 1 and *k*_B_*T* = 1 defines the internal unit of time as , where δ*t* = 0.01τ is the integration time step. Notably, the choice of the particle mass is arbitrary because it has no effect on the partition function, and hence, it does not affect any thermodynamic properties. It only affects dynamical properties of the system, which we did not study here.

#### 2.2.2 Reaction Steps—G-RxMC Monte Carlo

To sample the concentration of small ions, we use the Monte Carlo method in the Grand-reaction ensemble.^106^ We emulate the ion exchange between the PEC and supernatant using stochastic grand-canonical insertions and deletions of small ions, formally denoted by chemical reactions
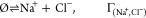
9
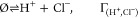
10
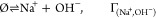
11
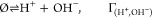
12

The equilibrium constants of these reactions are defined as
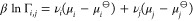
13where ν_*i*_ is the stoichiometric coefficient, μ_*i*_ is the chemical potential, and μ_*i*_^⊖^ is the standard chemical potential of species *i*. The constant  is the ionic product of water. Out of the remaining three equilibrium constants, , , and , two are linearly independent and can be chosen independently as input parameters which define the salt concentration and pH of the supernatant solution. The third constant cannot be chosen independently because its value is determined by the electroneutrality constraint (see Section S1.2). The exact salt concentration and pH in the supernatant are determined from an auxiliary simulation using the chosen values of equilibrium constants. Therefore, the reported values of the salt concentration usually are not round numbers, as expounded in ref (106).

When considering a weak diprotic acid as an additional solute, we add acid–base dissociation reactions of the solute to the above set of reactions
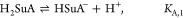
14
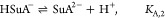
15where *K*_A,1_ and *K*_A,2_ are the respective acidity constants. For simplicity, we refer to this acid as succinic acid and accordingly, we use p*K*_A,1_ = 4.2 and p*K*_A,2_ = 5.6.^125^ To account for exchange of the solute between the supernatant and PEC, we add further reactions, analogical to the above
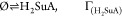
16
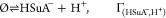
17
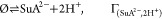
18

The equilibrium constants of the last three equations are determined by a combination of the acidity constants of the solute and its concentration in the supernatant, which we explain in detail in Section S1.2 of the Supporting Information.

To perform a single Monte Carlo step, we first randomly select one of the considered reactions, each of them with an equal probability. Next, we choose whether to carry out the reaction in the forward or backward direction, also with equal probabilities. For a chosen direction, a trial reaction move is carried out by removing particles, by changing the particle identities, inserting new particles at random positions, or deleting old ones, as prescribed by the stoichiometry of the corresponding reaction. The proposed new trial state (n) is accepted with the probability
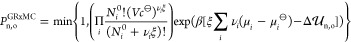
19Otherwise the original state (o) is kept. In eq 19, *V* denotes the box volume, ξ = ± 1 denotes the extent of the reaction, *c*^⊖^ = 1 mol/L denotes the reference concentration,  denotes the change in energy between the new state (n) and the original one (o), and finally, *N*_*i*_^0^ denotes the initial number of particles of type *i*.

Ultimately, the input of the method is the set of equilibrium constants of all reactions, Γ. The output is the composition of the system, determined by the average concentration of the exchangeable species in the simulation box. One can perceive this method as a combination of Monte Carlo in reaction ensemble and semigrand canonical ensemble, where we fix the linear combinations of chemical potentials of species because the chemical potentials of individual ionic species are ill-defined.^106^

#### 2.2.3 Preparation, Equilibration, and Production

Finally, we describe how we prepare the PEC phase. First, we initialize the polymers as fully extended rods regularly distributed on a primitive cubic lattice. We tune the relative accuracy of the PPPM to 10^–2^, and then we let the system thermalize by running 10^5^ steps of Langevin dynamics (10^3^τ), roughly corresponding to the Rouse time of an ideal chain with the same number of segments as our polyelectrolytes. Next, we gradually compress the box by setting the new box size in each dimension to *L* → *L* – Δ*L*, where Δ*L* = 0.7σ, and simultaneously, we rescale the Cartesian coordinates of all particles by the factor 1 – Δ*L*/*L*. The system is then anew relaxed by running 10^5^ steps of Langevin dynamics. The relaxation and compression are iteratively repeated until we reach the final value of *L*, defined by the desired mean polymer volume fraction
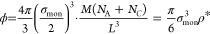
20which is our input parameter. Our typical volume fractions ϕ ≈ 15% correspond to the reduced particle densities ρ^*^σ^3^ ≈ 0.2 and box sizes *L* ≈ 30σ ≈ 7*R*_g_, where *R*_g_ is the radius of gyration of a single chain. These volume fractions are still low enough to ensure that the chains do not interact with their own periodic images through the boundary conditions. Upon reaching the final box size, we eventually retune the parameters of the PPPM method to the relative accuracy of 10^–3^, and we equilibrate the system for 10^7^ steps of Langevin dynamics (10^5^τ) and 10^5^ steps of Monte Carlo particle exchanges until time drifts in energies and pressures cease to be measurable. The subsequent production stage is typically 10^7^δ*t* = 10^5^τ long and every 10^4^δ*t* = 10^2^τ, we attempt 5 × 10^2^ Monte Carlo steps, adding up to 5 × 10^5^ Monte Carlo steps over the course of the simulation. To ensure that our systems were given sufficient time to relax, we estimated the relaxation times from the mean squared displacement of the chain centers of mass, as shown in Section S2.2 in the Supporting Information, demonstrating that the equilibration and production are significantly longer than Rouse time. Length of the simulations of the reservoir is the same as for the PEC phase.

### 2.3 Experimental Details

#### 2.3.1 Materials and PEC Formation

PAH (150 kDa) solution was purchased from Nittobo. All other reagents: PAA (100 kDa) acidic form solution, CoCl_2_, NaCl, HCl, and NaOH were purchased from Merck. PAH and PAA stock solutions were diluted to 80 g/L working solutions, and the pH was set to 7 with diluted HCl and NaOH solutions. Working solutions of PAH and PAA were mixed with salts in the following order of addition: water, NaCl, CoCl_2_, PAH, and then PAA.

Equal amounts of PAA and PAH monomers were added and thus also equal amounts of charge. The total sum of the PAA and PAH concentrations was kept constant at 2 g/L. The CoCl_2_ concentration was fixed at 1.7 mM, and NaCl concentrations were varied between 1 and 175 mM. Systems were left to equilibrate overnight before being centrifuged at 12,400*g* for 30 min to clearly separate the PEC phase from the supernatant phase.

#### 2.3.2 Estimation of Partition Coefficients and Water Content

The mass of the systems in vials was weighed at several points: empty, filled with all components, with the supernatant removed, and dried. By comparing the mass of the systems with the supernatant removed and when dried, we obtain the water content of the PEC. Drying is verified via evaluation of the cobalt color; hydrated wet cobalt is bright pink, while dehydrated cobalt is dark blue. Sufficient cobalt was present after drying to visually verify successful dehydration.

The concentration of Co^2+^ ions in the supernatant phase is determined using a Shimadzu 2401PC spectrophotometer via analysis of the absorbance at a wavelength of 510 nm. The Co^2+^ concentrations in the supernatant are compared to a polyelectrolyte-absent control with the same initial CoCl_2_ concentration. The partitioning coefficient *K*_Co_^2+^ is then determined as
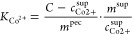
21where  is the concentration of Co^2+^ in the supernatant, normalized for the polyelectrolyte-absent control *C*, which is defined as 100%. The mass of the hydrated complex is *m*^pec^, while *m*^sup^ is the mass of the supernatant. An assumption was made that the density of (hydrated) PEC was approximately equal to that of water.^40^

## 3 Results

### 3.1 Determining Phase Coexistence

**Figure 2:**
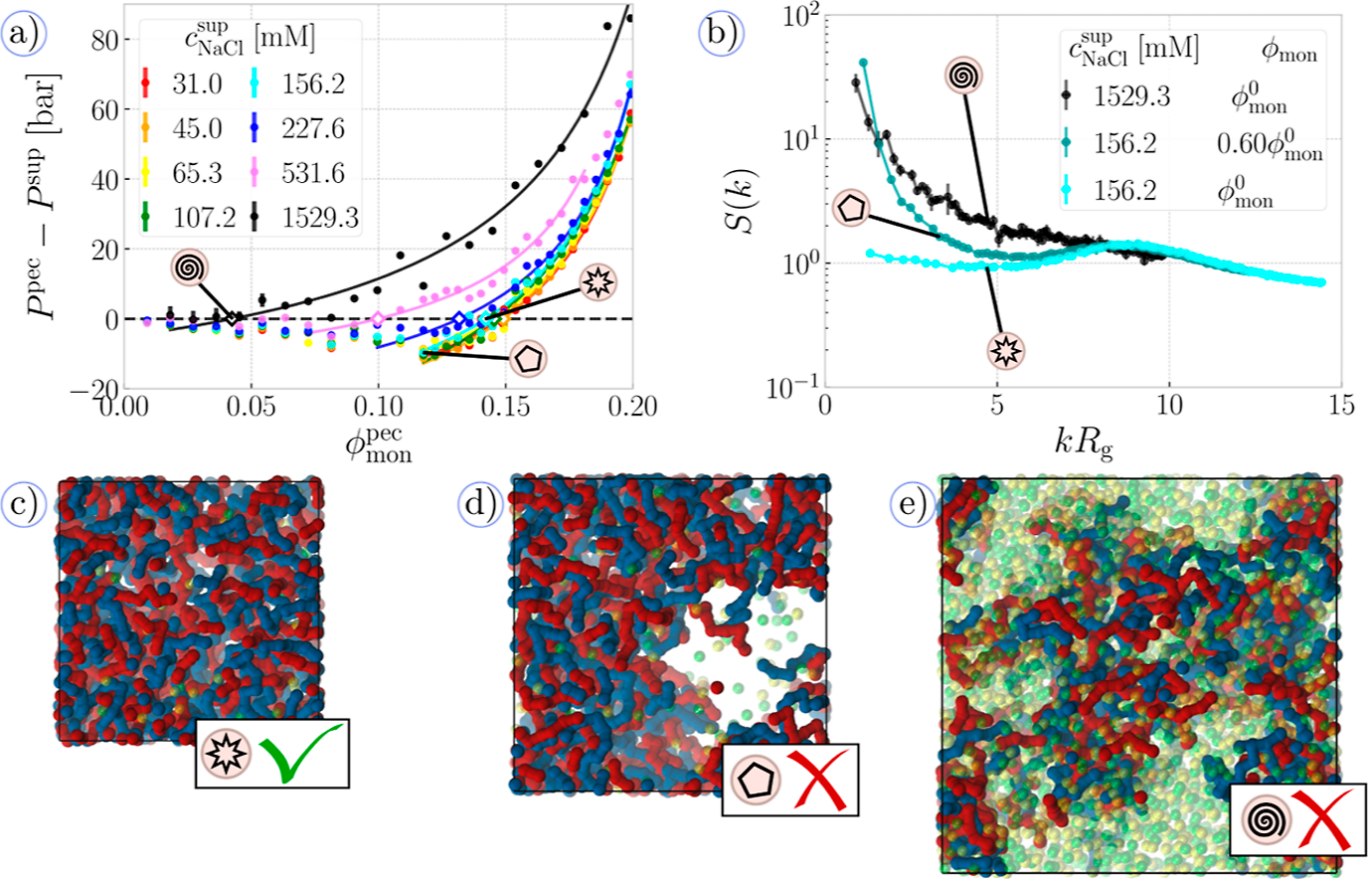
Pressure–composition protocol: (a) Difference between the pressure of the PEC phase and the NaCl supernatant as a function of monomer volume fraction in the PEC phase. Circles are data from simulations at different salt concentrations, *c*_NaCl_, and lines are fits discussed in Section 3.1. Hollow diamonds mark the monomer volume fractions in the PEC, which has the same osmotic pressure as the supernatant. Three black symbols in beige circles mark three selected systems shown in the snapshots below. (b) Static structure factor of monomer–monomer (A^–^ – A^–^) as a function of wavevector *k*, multiplied by radius of gyration of a single polyanion chain, plotted for the three selected compositions shown in the snapshots. (c–e) Snapshots of the three selected systems, using the same color as shown in Figure 1. Green ticks mark a stable single-phase system, whereas red crosses mark systems with undesirable phase separation within the box.

To determine the composition of the coexisting phases, we expand on the approach used in our previous simulations of polyelectrolyte gels.^112,121,126^ First, we select a supernatant phase of a given composition and then try to identify whether there could be an PEC phase in equilibrium with this supernatant. For such an PEC phase, at least two conditions have to be met: (i) chemical potentials of exchanged ions and solutes are equal in both of the phases, and (ii) pressures of both of the phases are equal. The first condition is automatically fulfilled by the G-RxMC algorithm.^106^ To meet the second condition, we utilize the pressure–composition protocol.^126^ We carry out a series of simulations of the PEC phase at various polymer volume fractions, determined by setting the simulation box length. In each simulation, we measure the osmotic pressure difference between the PEC and the supernatant, Δ*P*(ϕ_mon_) = *P*^pec^(ϕ_mon_) – *P*^sup^, as a function of the polymer volume fraction. Then, we fit the data using a smooth function to find the polymer volume fraction, ϕ_mon_^0^, such that Δ*P*(ϕ_mon_^0^) ≈ 0, as illustrated in Figure 2a.

A typical example of the pressure–composition protocol is represented by the cyan data set in Figure 2a, corresponding to *c*_NaCl_^sup^ ≈ 156 mM. We observe that Δ*P*(ϕ_mon_) ≫ 0 at high polymer volume fractions, mainly due to the steric repulsion. As the density of the PEC phase decreases, the Δ*P*(ϕ_mon_) dependence eventually crosses the zero-pressure baseline, indicating that the conditions for phase coexistence of supernatant and PEC can be fulfilled at a certain volume fraction, ϕ_mon_^0^, as shown on the snapshot in Figure 2c. While volume fractions at ϕ_mon_ > ϕ_mon_^0^ correspond to the states of PEC under compression, we have to be cautious about interpreting the compositions of ϕ_mon_ < ϕ_mon_^0^ because they no longer correspond to a single homogeneous phase. At ϕ_mon_ < ϕ_mon_^0^, the system can undergo a phase separation within the simulation cell, forming a set of polymer-rich and polymer-poor domains, as exemplified in Figure 2d. In such cases, the actual monomer density of the polymer-rich domains is not equal to the mean monomer density. Naturally, such a separation within the box is affected by massive finite-size effects and leads to large fluctuations of pressure. Therefore, we fit the *P*(ϕ_mon_) dependence only in the range where Δ*P* ≳ 0, in order to avoid the region of possible phase separation and simultaneously obtain a reliable estimate of ϕ^0^. We employ a phenomenological fitting function, Δ*P*(ϕ) = *a*_0_ + *a*_1_/tan(ϕ – *a*_2_) ,where *a*_0_, *a*_1_, and *a*_2_ are the adjustable parameters. This function is monotonic and qualitatively accounts for the shape and curvature of the *P*(ϕ_mon_) dependence, as explained in ref (121).

The final step of the protocol is the assessment of self-consistency. As noted above, we require the PEC at ϕ_mon_^0^ to be a homogeneous single-phase system. For that reason, we analyze the spherically averaged structure factors, *S*(*k*), between monomeric units, as displayed in Figure 2b. If the system should demix to form polymer-rich and polymer-poor domains, the structure factor would diverge in the limit of small wavevectors, *k* → 0.^114^ In Figure 2b, for *c*_NaCl_ ≈ 156 mM we indeed see such a demixing peak in *S*(*k*) of the system with mean volume fraction 0.60ϕ_mon_^0^, indicating the separation. On the contrary, *S*(*k*) of the PEC with mean volume fraction of ϕ_mon_^0^ does not feature such a peak, indicating a stable homogeneous phase, coexisting with the supernatant. We can observe similar hallmarks of demixing at very high salt concentrations, *c*_NaCl_ ≈ 1529 mM, as testified by the peak in Figure 2b and visual inspection of the snapshot in Figure 2e. The hallmarks of demixing were observed also in the point at ϕ_mon_ ≈ 0.08, appearing close to Δ*P* = 0. Because the condition of phase stability was not satisfied in the above cases, we conclude that our simulations indicate a stable PEC phase coexisting in equilibrium with the supernatant only at *c*_NaCl_ < 1529 mM. At higher salt concentrations, one should observe formation of finite-size aggregates but not macroscopic phase separation.

Despite the above qualitative analysis, we emphasize that our approach cannot properly identify the critical point because the assumption of no polymer in the supernatant phase breaks down in that region. Furthermore, as shown in Section S2.1 in the Supporting Information, we see that in our finite simulation box the scattering peak at *k* ≈ 0 gradually emerges as *c*_NaCl_ is increased. Therefore, we need to introduce an arbitrary discrimination criterion as a limit of validity of our approximations. In our case, we consider the system to be demixing if the peak at *k* ≈ 0 is the global maximum of the *S*(*k*) curve. Accordingly, as the *c*_NaCl_ increases, so does also the systematic error in our approach to identifying phase coexistence. Nevertheless, constructing the full phase diagrams is not our objective. Instead, we focus on the two-phase regions far from the critical point, where our method can capture ample features of the experimental systems, as will be demonstrated in the following subsections.

### 3.2 Phase Diagrams and Partitioning of Small Ions: Simulation vs Experiment

**Figure 3:**
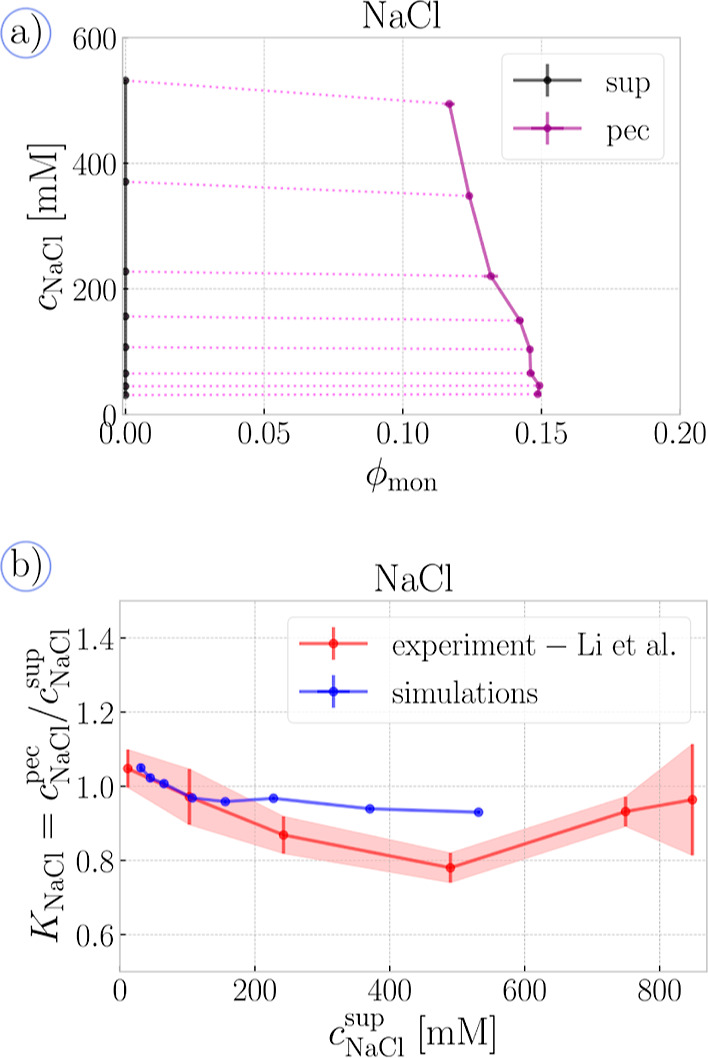
Simulations vs experiment (monovalent): (a) phase diagram of PEC coupled to the supernatant containing only NaCl ions, in the plane of monomer volume fraction, ϕ_mon_ vs salt concentration, *c*_NaCl_. The black line shows the compositions of the supernatant phase (^sup^), whereas the purple line shows the corresponding PEC phase (^pec^), connected by a violet tie-line. (b) Partition coefficient of NaCl between the phases as a function of salt concentration in the supernatant. The experimental data for PLK_50_ – PRE_50_ have been digitized from Figure 5 of ref (86)

In Figure 3a, we plot a phase diagram, where we used the pressure–composition protocol from Figure 2 to relate the composition of the supernatant and the coexisting PECs. The phase diagram manifests some typical features of polyelectrolyte complexation. First, the polymer volume fraction determined from our simulations is between ∼10% and ∼15%, which is within the rather broad range reported by various experiments and simulations.^5^ Next, the PEC density decreases with increasing *c*_NaCl_, as expected due to stronger electrostatic screening. This works well far enough from the critical point when it is reasonable to assume that there is no polymer in the supernatant. However, our model cannot accurately predict that the PEC eventually dissolves when the critical *c*_NaCl_ is reached. Nevertheless, our predictions of the phase coexistence remained self-consistent up to *c*_NaCl_ ≈ 532 mM. Accordingly, the critical salt concentration would be greater than 532 mM, which is consistent with critical point reported in various studies, anywhere between 500 and 3000 mM, depending on specific parameters of the systems.^5^ Finally, in Figure 3b, we show that partition coefficients of NaCl between the two phases, obtained by dividing the salt concentrations at the end-points of the tie-lines in Figure 3, are commensurate with partition coefficients in PECs formed by poly(l-lysine)/poly(d,l-glutamic acid) polypeptides of length 50, measured in ref (86). The experimental data span a broader concentration range, including the critical region, which is inaccessible to our simulations. In the limit of low *c*_NaCl_, the partition ratio is slightly above unity, but it becomes smaller than one if *c*_NaCl_ is increased, pointing to the preferential partitioning of monovalent ions into the supernatant phase. This behavior is in agreement not only with experiments but also with recent liquid-state theories,^60,67,77^ which attribute it to steric repulsion and correlations due to the chain connectivity, both of which are explicitly included in our simulation model. The above studies also observe that at high *c*_NaCl_, the partition ratio starts to increase and approaches unity close to the critical point. We do not observe this regime as our model loses validity close to the critical point.

**Figure 4:**
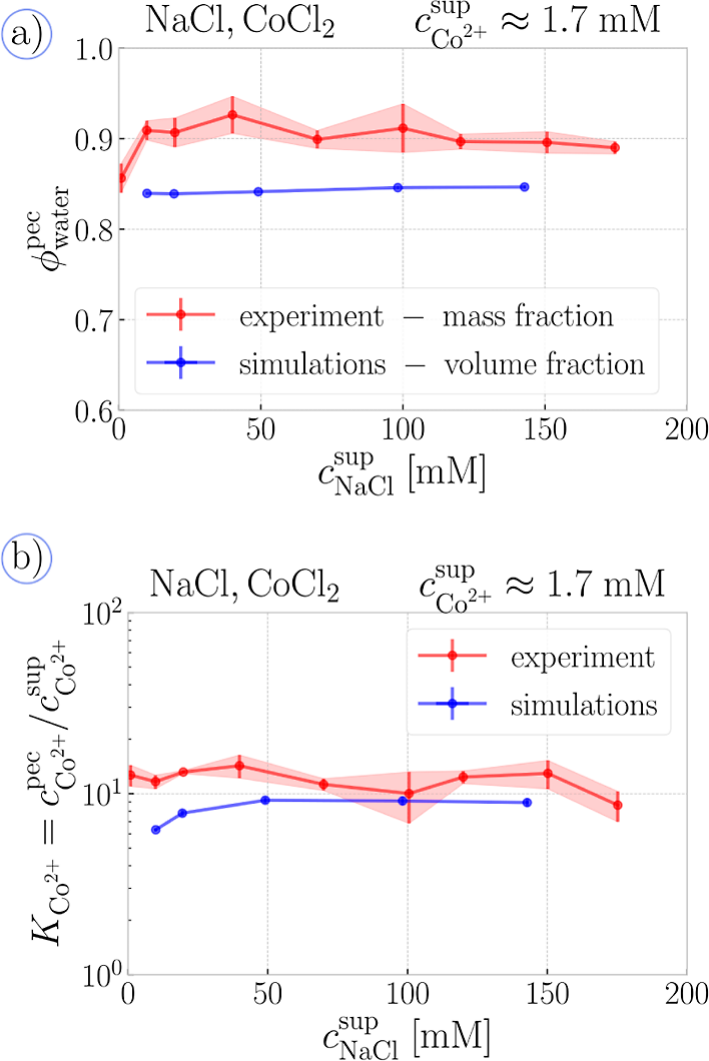
Simulations vs experiment (divalent): (a) water content of the PEC phase as a function of the salt concentration in the supernatant compared for the volume fraction from the simulations and mass fraction from our thermogravimetric experiments. (b) Partition coefficient of CoCl_2_ between the phases as a function of the salt concentration in the supernatant. The error bands in all panels show the estimated error of the mean.

In Figure 4, we explore complexes coexisting with the supernatant containing a mixture of NaCl and CoCl_2_, comparing the simulations with our own experiments. First, in Figure 4a, we see that the water content in these complexes only weakly depends on *c*_NaCl_. In the experiment, the water content is determined from the mass fraction, which is readily available by dividing the mass of wet complex and dried complex (Figure S8), showing that water comprises ∼90% of the complex. In our simulations, the mass fraction is not well-defined because we are using an implicit solvent. Therefore, we used volume fractions of the polymer, defined in eq 20, and volume fraction of ions, ϕ_ion_, defined in analogy to the polymer fraction. Then the volume fraction of water in the PEC phase is calculated as ϕ_water_^pec^ = 1 – ϕ_mon_^pec^ – ϕ_ion_^pec^, which can be assumed proportional to the mass fraction of water by a multiplicative factor, given by the ratio of mean mass densities of water and the wet complex. As the wet complex is mostly composed of water, the above factor should be close to unity, although its accurate value cannot be determined. The use of an implicit solvent model adds an additional uncertainty in comparing the volume fractions from simulations with mass fractions from experiments. Namely, the effective particle size indirectly includes the effect of solvent molecules; for example, the effective size of ions includes their first hydration shell. Given this inherent uncertainty, we interpret the comparison of partition coefficients in Figure 4 as a sufficient concurrence between the experiment and simulation. We reiterate that absolute quantitative agreement between the two can be, in principle, reached by fine-tuning of model parameters, but this is not the main objective of our study. We will comment on this point in more detail in Section 3.3. Finally, the experiments also show that the ratio between the polymer concentrations in the PEC and supernatant is ≈200 (Section S3 in the Supporting Information), hence validating our assumption of near-to-none polymer in the supernatant.

Figure 4b reveals that divalent ions preferentially accumulate in the PEC phase. Experiments show a partition coefficient (*K*_Co_^2+^ ≈ 12), virtually independent of *c*_NaCl_, while simulations suggest a partition coefficient of *K*_Co^2+^_ ≈ 6 in the low-salt limit, followed by a slight increase up to plateau of *K*_Co^2+^_ ≈ 9 as *c*_NaCl_ increases. These results are commensurate with experiments of Iyer et al.^88^ who measured partition coefficients of CaCl_2_ in PAA–PAH complexes within the range 3–10 at ionic strengths between 0.2 and 2 M. Interestingly, the transfer matrix calculations and Monte Carlo simulations in ref (73) report salt MCl_2_ to prefer the supernatant. The reasons for this discrepancy are not clear to us at the moment; however, one possible explanation is that they used a slightly weaker electrostatic coupling, σ/*l*_B_ ≈ 1.6 and different radius of salt ions. Nevertheless, the matching order of magnitude of partition coefficients of various multivalent ions, CaCl_2_ and SrCl_2_ used in ref (88) and also CoCl_2_ used in ours, underpins the general effect of dominant contribution of valency and Coulombic interactions in the partitioning of the multivalent ions. Albeit the ion-specific effects, known in the context of Hoffmeister series, certainly can play a role^83,127,128^ in experimental systems, we emphasize that in our simulations, the only difference between Co^2+^ and Na^+^ ions is their charge, while all the other interactions are kept the same. Therefore, our simulations clearly show that a change in the valency alone is sufficient to ensure preferential accumulation of multivalent ions in the PEC phase, yielding almost quantitative agreement with the experiments.

### 3.3 Sequestration of Weak Diprotic Acid

To demonstrate the capabilities of our modeling approach, we use it to calculate the partitioning of a weak diprotic acid, i.e., a small solute that can undergo charge regulation. The charge states of this acid depend on the pH and are further modulated by intermolecular interactions, in particular electrostatics. Our model of the PEC contains equal amounts of polycations and polyanions; therefore, the Donnan potential between the complex and the supernatant phase is zero, causing the pH in both phases to be the same. However, the ionic strength in the PEC phase is much higher than that in the supernatant. According to the Debye–Hückel theory, an increased ionic strength decreases the activity coefficients of charged species, causing charged states of weak acids to be more preferred than the uncharged ones. Although the Debye–Hückel theory is not quantitatively correct under these conditions, the qualitative explanation remains valid. Simultaneously, permittivity in the interpolyelectrolyte phase is slightly lower than that in the supernatant. Both these effects synergistically cause that the electrostatic contribution to the free energy is more favorable in the PEC phase. Consequently, charged states of the solute should be preferred in the PEC, much more than in the supernatant.

**Figure 5:**
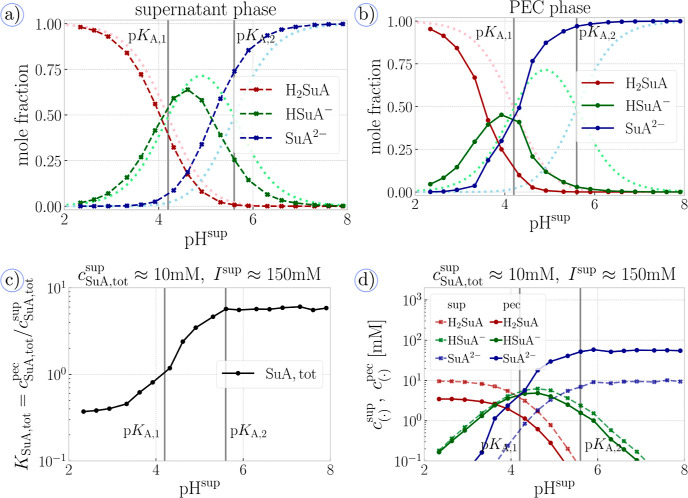
Partitioning of small acid: (a) ionization response of succinic acid in the supernatant phase at *c*_SuA,tot_^sup^ ≈ 10 mM and NaCl at total ionic strength *I*^sup^ ≈ 150 mM, dark symbols and lines are from simulations, light dotted lines correspond to the Henderson–Hasselbalch equation (ideal system). (b) Ionization response of succinic acid in the PEC phase coexisting with the supernatant in panel (a); dark symbols and lines are from simulations, and light dotted lines correspond to the Henderson–Hasselbalch equation. (c) Partition coefficient of all ionic forms of succinic acid between the phases as a function of pH at *c*_SuA,tot_^sup^ ≈ 10 mM and NaCl at total ionic strength *I*^sup^ ≈ 150 mM. (d) Concentration of all ionic forms of the succinic acid as a function of pH, displayed for both supernatant phase (dashed lines) and PEC phase (solid lines).

In Figure 5, we compare the populations of different charge states of the weak acid in both supernatant and PEC. As a reference, we use the Henderson–Hasselbalch equation, which neglects all interactions and predicts the ionization state in the ideal limit. In Figure 5a, we observe that the population of the uncharged state, H_2_SuA, in the supernatant phase is slightly suppressed, as compared to the ideal result at pH ≈ 4 ≈ p*K*_A,1_. At this pH, the monovalent state, HSuA^–^, is favored more than the uncharged one. The population of the monovalent state reaches a maximum at a pH slightly lower than the maximum of the ideal curve, which is at pH = (p*K*_A,1_ + p*K*_A,2_)/2. At pH ≈ 5.5 ≈ p*K*_A,2_, we observe that the population of the monovalent state is significantly suppressed in favor of the divalent state, SuA^2–^. These differences can be alternatively viewed as a shift of the ideal curves to lower pH values (dashed lines in Figure 5a). According to the Debye–Hückel theory (see Section S2.4), this shift should be proportional to valency of the respective ion. However, our simulations show that the population of the divalent species is shifted by about 0.3 units of the pH, more than twice as much as the population of the monovalent one, demonstrating the nonlinear effect of electrostatic correlations.

The nonlinear effect of electrostatic correlations gets further amplified when we examine the charge states of the same solute in the PEC phase, as shown in Figure 5b. Here, we observe that population of the uncharged state is suppressed much more than that in the supernatant, and it is shifted to lower pH values by about 0.5 unit. Population of the monovalent state reaches a maximum at pH < 4 < p*K*_A,1_, which is more than 1 pH unit lower than position of the maximum of the ideal curve. Population of the divalent ions starts to dominate already at pH ≈ 4, although the ideal curve predicts that this population should be vanishingly small at the given pH. Again, the nonlinear effect of electrostatic correlations causes the divalent state of the diprotic acid to be strongly preferred in the PEC and starts to dominate already two units of pH below the value p*K*_A,2_. Our arguments given above are supported by experimental evidence and independent theoretical analysis by Choi et al.,^91^ published while our manuscript was under review. They monitored the ionization states of a fluorescent pH indicator, observing that its more ionized form (*z* = −3) is more abundant in the PEC phase than in the supernatant at one specific pH value. They interpreted this observation as a shift in the effective p*K*_A_ value as a result of favorable electrostatic interactions with the PEC phase.

The strong preference of ionized states in the PEC affects not only their populations within this phase but also the overall partitioning of diprotic acid between the two phases. In Figure 5c, we show that the partition coefficient of the diprotic acid, *K*_SuA,tot_, strongly increases as a function of the pH. At low pH values, when the uncharged state dominates, the partitioning is governed by the steric repulsion, resulting in *K*_SuA,tot_ < 1. As soon as the monovalent state starts to dominate, we observe that *K*_SuA,tot_ > 1, as a consequence of electrostatic interactions. As the pH is further increased, *K*_SuA,tot_ continues to increase until it saturates at a value of *K*_SuA,tot_ ≈ 5, which is slightly lower than the partition coefficients of divalent ions observed in Section 3.2. This difference can be explained by the concentration of the diprotic acid being about six times higher than the concentration of the divalent salt in Section 3.2. This higher concentration was required to ensure good statistics on partitioning of individual ionization states. As a side effect, it caused that the fully ionized diprotic acid significantly affects the ionic strength in the supernatant and thereby also the activity coefficients of all other ions.

To further elucidate the complexity of partitioning of multiprotic solutes, we plot in Figure 5d the concentrations of various ionized states of the diprotic acid in the supernatant. This figure shows that the concentration of the uncharged state in the complex is about two times lower than that in the supernatant and in PEC. This is because the interaction of the uncharged species is dominated by the steric repulsion which favors the more dilute supernatant over the highly concentrated PEC. In contrast, the concentrations of monovalent species are almost the same in both supernatant and complex phase with a slight preference of the supernatant phase, in agreement with the partitioning of monovalent salt, discussed in Section 3.2. Finally, the concentration of divalent species is about six times higher in the complex phase than in the supernatant, in agreement with the partitioning of divalent salt. Thus, we can conclude that partitioning of a diprotic acid as a function of pH represents a gradual transition from the behavior dominated by steric repulsion to that dominated by electrostatic interactions. However, this transition is strongly affected by deviations from the ideal behavior; therefore, it cannot be correctly described by the ideal model of acid–base ionization.

### 3.4 Sensitivity of Our Results to the Choice of Model Parameters

In the preceding section, we showed that our generic coarse-grained model qualitatively mirrors multiple features of PEC phase separation, observed in experiments and predicted by other computational and theoretical models. Minor quantitative discrepancies between various experiments and theoretical models can be explained by differences in chemical details, such as the size of the monomeric units, permittivity of the PEC phase, charge density on the polymer chains, or specific polymer–polymer and polymer–solvent interactions. However, systematic and independent variation of these parameters is impossible in experimental systems or in models that have been tuned to reproduce one specific experimental system. In this section, we exploit the feature of our generic model that allows us to vary these parameters systematically in order to explore how a quantitative agreement between simulation and experiment can be reached by tuning of the model parameters. Furthermore, it allows us to distinguish generic features of the PEC phase separation from those that depend on specific details of the model.

#### 3.4.1 Monomer Bulkiness and Charge Density

The bulkiness of the monomeric units and the asymmetry in size between the monomeric units and small ions affect the balance between steric repulsion and electrostatic interactions. The electrostatic interaction provides a cohesive force which favors phase separation, whereas the steric repulsion has the opposite effect. Therefore, the choice of particle sizes in the simulation model affects the location of the phase boundaries in the phase diagram.

We chose the size of the monomeric units and small ions within a range commonly used in coarse-grained simulations of similar systems. For instance, the authors of ref (129) proposed a bottom-up coarse-graining of PAA from atomistic simulations, where distances between different carbon atoms between neighboring PAA groups ranged from 0.250 to 0.450 nm. Herein, we chose σ_mon_ ≈ 0.426 nm = 1.20σ to represent an effective size of whole PAA monomeric unit, modeled by a single particle. For simplicity, we chose the same size for the polycation, using the monomer–monomer distance from the coarse-grained model of PAH proposed in ref (130). For the size of the ions, we used a slightly smaller value, σ_ion_ = 0.355 nm, matching our previous work,^126^ where we demonstrated, that this ion size reproduces the experimentally determined activity coefficients in aqueous solutions of NaCl,^131^ in a broad range of concentrations, up to the solubility limit of *c*_NaCl_ ≈ 700 mmol/L.

**Figure 6:**
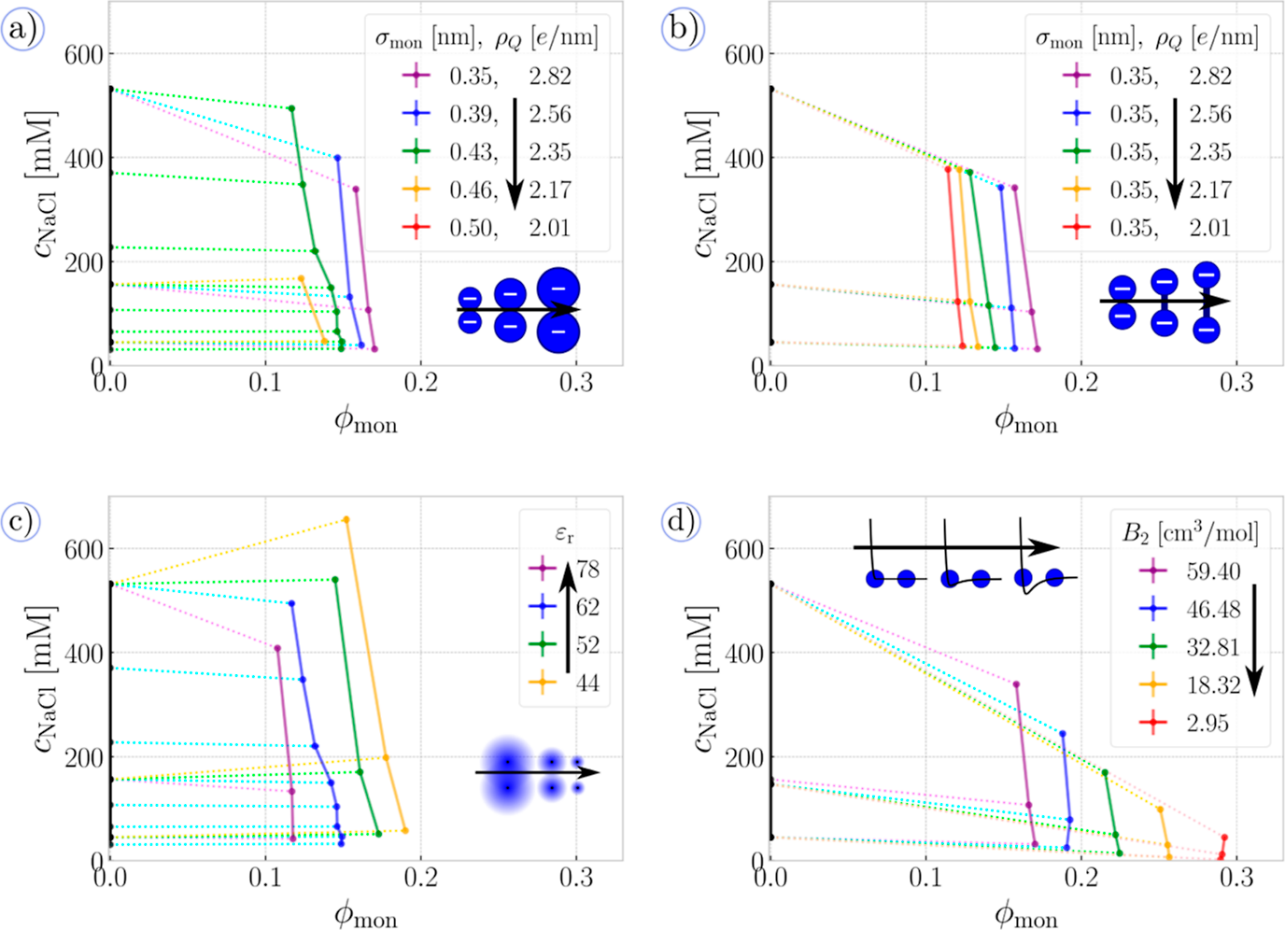
Sensitivity to parameters: the effect of varying model parameters on the phase diagrams for PEC coupled to the supernatant containing only NaCl. The insets schematically depict which parameters are being varied. (a) Simultaneous variation of both bulkiness of monomeric units, σ_mon_, and charge density, ρ_Q_, (b) variation of charge density only, ρ_Q_, (c) variation of relative permittivity of the polyelectrolyte phase, ε_r_, and (d) variation of the solvent quality, quantified by the second virial coefficient, *B*_2_.

In Figure 6a, we explore the effect of the monomer bulkiness on the phase diagram of PEC in equilibrium with a supernatant containing only NaCl. Because our excluded-volume potential is much steeper at small distances than the bonding potential, an increase in the size of the particle representing the monomeric units, σ_mon_, implies a simultaneous increase in the mean bond length to ⟨*b*⟩ ≈ σ_mon_, thereby decreasing the effective charge density along the chain contour. Lowering the charge density on the polymer decreases the entropic gain from the release of counterions and suppresses the electrostatic interactions between the chains, thereby destabilizing the PEC phase. This decrease acts in synergy with an increased range of steric repulsion. Consequently, an increase in σ_mon_ shrinks the coexistence region. Notably, if we use σ_mon_ = 1.30σ ≈ 0.46 nm on Figure 6a, the pressure–composition protocol does not indicate a stable PEC phase at *c*_NaCl_ ≈ 532 mM, whereas a stable PEC phase exists at the same *c*_NaCl_ if we use σ_mon_ = 1.20σ ≈ 0.43 nm. Eventually, if we use σ_mon_ = 1.40σ ≈ 0.50 nm, there is no phase coexistence at all, and the phase diagram contains only a single-phase region.

In order to separate the synergistic effects of monomer bulkiness and charge density, in Figure 6b, we fixed the size of monomeric units to σ_mon_ = 1.00σ ≈ 0.35 nm, and we varied only the bond length, yielding the same range of charge densities as in Figure 6a while keeping the steric repulsion unchanged. Our results in Figure 6b show that a decrease in the charge density shrinks the phase separation region, as predicted by theories^72^ and observed in experiments.^132^

#### 3.4.2 Permittivity and Quality of the Solvent

Because of limited availability of suitable inputs, choosing the permittivity of the PEC phase is much more tricky than choosing the sizes of monomeric units or small ions.^133^ The supernatant phase is a solution of small ions is sufficiently dilute to assume that its permittivity is close to that of pure water, ε_r_ ≈ 78. However, the PEC phase should have a lower permittivity than pure water because of high polymer volume fraction, typically about ∼10%.^134^ This idea is further supported by ref.,^86^ where the authors used permittivity of ε_r_ ≈ 30 to match their simulations with experiments. In contrast, the simulations in ref (72) just used the permittivity of water, however, their approach was aimed at the strong coupling regime, where the variation of permittivity does not have a strong effect on the phase behavior.

In Figure 6c, we show phase diagrams obtained by varying the permittivity of the complex phase while keeping the permittivity of the supernatant at ε_r_ ≈ 78. A decrease in permittivity amplifies electrostatic interactions between all charged particles. The cohesive effect of electrostatics not only extends the two phase region but also causes preferential partitioning of NaCl into the phase of the complex at ε_r_ ≲ 52. This is in line with recent experiments and simulations which acknowledged that the partition coefficients of NaCl can indeed exceed unity if they use short-range attractions between ions and polymers.^132^ This, however, unveils a problem–our simulations and those in ref (132) both observe qualitatively the same trend in partition coefficients of NaCl but for different reasons. Even though our model has just a few tuneable parameters, it brings about a lot of flexibility, and one has to be careful when setting the model parameters to identify the correct source of phenomena with respect to the specific experiment and to avoid overfitting.

Similar to permittivity, another phenomenological parameter to set is the quality of the solvent, collectively describing effective interactions between the monomeric units as compared to the polymer–solvent interactions. We characterize these interaction using the second virial coefficient, *B*_2_, as explained in Section S1.1 in the Supporting Information. The value of *B*_2_ is the highest for purely repulsive interaction (athermal solvent), and it gradually decreases toward the θ-state, which is defined by *B*_2_ ≈ 0. Clearly, additional attraction between the monomeric units further stabilizes the coacervate phase, extending the coexistence region, in agreement with ref (82). Moreover, we observe significant decrease in the partition coefficient of NaCl as quality of solvent drops since the phase of the complex becomes more crowded.

#### 3.4.3 Size of the Simulated System

The finite size of the simulated system may have a significant impact on the location of phase boundaries determined from a simulation with periodic boundary conditions.^135^ Only in the limit of infinite system size do such simulations converge to the properties of bulk phase-separated systems. To demonstrate that our results were not significantly affected by the size of the simulated system, in Section S2.3 in the Supporting Information we present structural analysis of systems with various sizes, determined by the numbers of polymer chains in the PEC, *N*_A_ = *N*_C_, ranging from 32 up to 96. In a typical simulation, we observe that *L* ≈ 7*R*_g_, where *R*_g_ is the mean radius of gyration of the polyions. Therefore, the box is large enough, so that chains do not interact strongly with their own periodic images. Accordingly, we observe in Section S2.3 that the positions of binodals and partition coefficients of the ions are virtually the same for all simulated sizes of the system. Therefore, we used *N*_A_ = *N*_C_ = 32 in all further simulations, which had the most favorable computational costs.

## 4 Conclusions

We have shown that a coarse-grained simulation model, combined with the grand-reaction method for simulating acid–base equilibria in two-phase systems, can predict the phase stability of PEC and the partitioning of ionic solutes between the PEC phase and the supernatant. We used the pressure–composition protocol to determine the conditions under which the PEC phase coexists with the supernatant of a given composition. Furthermore, we have shown that the pressure–composition protocol should be supplemented by structural analysis of the PEC phase in order to ensure that phase separation does not occur within the simulation box, resulting in polymer-rich and polymer-poor domains.

Our model is suitable for predicting the phase coexistence far from the critical point. However, it cannot correctly describe the critical region because it assumes that no polymer is present in the supernatant phase. In accordance with the literature, our simulations predict that the stability window of the PEC phase shrinks as the salt concentration is increased. Furthermore, they predict that the partition coefficients of monovalent ions are slightly below unity, indicating a slight preference for the supernatant phase. Unlike monovalent ions, divalent ions strongly prefer the PEC phase, resulting in partition coefficients on the order of 10. These results semiquantitatively agree with our own experiments and also with other experiments from the literature.

Next, we have shown that the ionization of a weak diprotic acid is significantly enhanced in the PEC phase as compared to the supernatant. Therefore, the partitioning of such a weak acid between the two phases can be tuned by varying the pH. At low pH, the weak diprotic acid is uncharged; therefore, steric repulsion dominates its interactions, causing it to prefer the supernatant phase. As the pH is increased, the preference for the PEC phase increases concomitantly. Divalent form of the diprotic acid starts to dominate in the PEC phase already at pH values when only its neutral and single-ionized forms are found in the supernatant. Consequently, diprotic acid significantly accumulates in the PEC phase already at much lower pH values than could be expected from its ionization in the aqueous solution of the supernatant. The above theoretical predictions are supported by experimental observations and an independent theoretical analysis in a recent study by Choi et al.^91^ This observation demonstrates that the partitioning of weak multiprotic acids can be easily tuned by varying the pH, providing interesting opportunities for engineering applications, such as separation of small acids based on their p*K*_A_.

Finally, by systematically varying some parameters of the coarse-grained model, we have shown that the above observations are generic features of multivalent ions in coacervates rather than incidental results caused by a specific choice of model parameters. We determined how the model parameters affect the partition coefficients and shape of the phase diagram, particularly the region of phase coexistence. We showed that an increase in monomer size or a decrease in charge density both shrink the coexistence region, whereas a decrease in permittivity of the PEC phase or a decrease in solvent quality, quantified by the second virial coefficient *B*_2_, both expand the coexistence region. Simultaneously, charge density and solvent quality have only negligible impact on the partition coefficients of small ions, whereas the monomer size and permittivity of the PEC phase significantly affect the partition coefficients to such an extent that variation of these parameters may even switch the preferential accumulation of the ions from supernatant to the PEC phase. Thus, it should be possible to tune the simulation model in order to match an arbitrarily selected set of experimental results. Additionally, various combinations of these parameters can lead to nearly identical phase diagrams, producing a good match with the experiment. Therefore, even a quantitative agreement between simulations and experiments does not automatically imply that the model parameters were chosen correctly. Nonetheless, the generic patterns in the partitioning of multivalent ions described above may be quantitatively but not qualitatively affected by small changes in parameters of the model.

## Supporting Information Available

The following files are available free of charge. The Supporting Information is available free of charge at https://pubs.acs.org/doi/10.1021/acs.macromol.3c01209.

Details on the simulation methodology; additional simulation results; and selected raw experimental data (PDF)

## References

[ref1] Muthukumar M. 50th Anniversary Perspective: A Perspective on Polyelectrolyte Solutions. Macromolecules 2017, 50, 9528–9560. 10.1021/acs.macromol.7b01929. 29296029 PMC5746850

[ref2] Gucht J. v. d.; Spruijt E.; Lemmers M.; Cohen Stuart M. A. Polyelectrolyte complexes: Bulk phases and colloidal systems. J. Colloid Interface Sci. 2011, 361, 407–422. 10.1016/j.jcis.2011.05.080. 21705008

[ref3] Tiebackx F. W. Gleichzeitige Ausflockung zweier Kolloide. Z. Chem. Ind. Kolloide 1911, 8, 198–201. 10.1007/BF01503532.

[ref4] Overbeek J. T. G.; Voorn M. J. Phase separation in polyelectrolyte solutions. Theory of complex coacervation. J. Cell. Comp. Physiol. 1957, 49, 7–26. 10.1002/jcp.1030490404. 13449108

[ref5] Sing C. E.; Perry S. L. Recent progress in the science of complex coacervation. Soft Matter 2020, 16, 2885–2914. 10.1039/D0SM00001A. 32134099

[ref6] Rumyantsev A. M.; Jackson N. E.; de Pablo J. J. Polyelectrolyte Complex Coacervates: Recent Developments and New Frontiers. Annu. Rev. Condens. Matter Phys. 2021, 12, 155–176. 10.1146/annurev-conmatphys-042020-113457.

[ref7] Wasilewski T. Coacervates as a Modern Delivery System of Hand Dishwashing Liquids. J. Surfactants Deterg. 2010, 13, 513–520. 10.1007/s11743-010-1189-4.

[ref8] Schmitt C.; Turgeon S. L. Protein/polysaccharide complexes and coacervates in food systems. Adv. Colloid Interface Sci. 2011, 167, 63–70. 10.1016/j.cis.2010.10.001. 21056401

[ref9] Gruber D.; Ruiz-Agudo C.; Cölfen H. Cationic Coacervates: Novel Phosphate Ionic Reservoir for the Mineralization of Calcium Phosphates. ACS Biomater. Sci. Eng. 2023, 9, 1791–1795. 10.1021/acsbiomaterials.1c01090. 35061343

[ref10] Insua I.; Wilkinson A.; Fernandez-Trillo F. Polyion complex (PIC) particles: Preparation and biomedical applications. Eur. Polym. J. 2016, 81, 198–215. 10.1016/j.eurpolymj.2016.06.003. 27524831 PMC4973809

[ref11] Margossian K. O.; Brown M. U.; Emrick T.; Muthukumar M. Coacervation in polyzwitterion-polyelectrolyte systems and their potential applications for gastrointestinal drug delivery platforms. Nat. Commun. 2022, 13, 2250 10.1038/s41467-022-29851-y. 35474060 PMC9042848

[ref12] Blocher W. C.; Perry S. L. Complex coacervate-based materials for biomedicine. Wiley Interdiscip. Rev.: Nanomed. Nanobiotechnol. 2017, 9, e1442 10.1002/wnan.1442. 27813275

[ref13] Bediako J. K.; Kang J.-H.; Yun Y.-S.; Choi S.-H. Facile Processing of Polyelectrolyte Complexes for Immobilization of Heavy Metal Ions in Wastewater. ACS Appl. Polym. Mater. 2022, 4, 2346–2354. 10.1021/acsapm.1c01634.

[ref14] Valley B.; Jing B.; Ferreira M.; Zhu Y. Rapid and Efficient Coacervate Extraction of Cationic Industrial Dyes from Wastewater. ACS Appl. Mater. Interfaces 2019, 11, 7472–7478. 10.1021/acsami.8b21674. 30689337

[ref15] Zhang Z.; Liu Q.; Sun Z.; Phillips B. K.; Wang Z.; Al-Hashimi M.; Fang L.; Olson M. A. Poly-Lipoic Ester-Based Coacervates for the Efficient Removal of Organic Pollutants from Water and Increased Point-of-Use Versatility. Chem. Mater. 2019, 31, 4405–4417. 10.1021/acs.chemmater.9b00725.

[ref16] Sproncken C. C. M.; Gumí-Audenis B.; Foroutanparsa S.; Magana J. R.; Voets I. K. Controlling the Formation of Polyelectrolyte Complex Nanoparticles Using Programmable pH Reactions. Macromolecules 2023, 56, 226–233. 10.1021/acs.macromol.2c01431. 36644553 PMC9835975

[ref17] Huang Y.; Lawrence P. G.; Lapitsky Y. Self-Assembly of Stiff, Adhesive and Self-Healing Gels from Common Polyelectrolytes. Langmuir 2014, 30, 7771–7777. 10.1021/la404606y. 24476067

[ref18] Yang M.; Digby Z. A.; Chen Y.; Schlenoff J. B. Valence-induced jumps in coacervate properties. Sci. Adv. 2022, 8, eabm4783 10.1126/sciadv.abm4783. 35584213 PMC9116606

[ref19] Lemmers M.; Spruijt E.; Akerboom S.; Voets I. K.; van Aelst A. C.; Cohen Stuart M. A.; van der Gucht J. Physical Gels Based on Charge-Driven Bridging of Nanoparticles by Triblock Copolymers. Langmuir 2012, 28, 12311–12318. 10.1021/la301917e. 22834713

[ref20] Voets I. K.; de Keizer A.; Cohen Stuart M. A. Complex coacervate core micelles. Adv. Colloid Interface Sci. 2009, 147–148, 300–318. 10.1016/j.cis.2008.09.012. 19038373

[ref21] Lemmers M.; Sprakel J.; Voets I. K.; van der Gucht J.; Cohen Stuart M. Multiresponsive Reversible Gels Based on Charge-Driven Assembly. Angew. Chem., Int. Ed. 2010, 49, 708–711. 10.1002/anie.200905515. 20017174

[ref22] Gao; Holkar; Srivastava Protein-Polyelectrolyte Complexes and Micellar Assemblies. Polymers 2019, 11, 1097 10.3390/polym11071097. 31261765 PMC6680422

[ref23] Water J. J.; Schack M. M.; Velazquez-Campoy A.; Maltesen M. J.; van de Weert M.; Jorgensen L. Complex coacervates of hyaluronic acid and lysozyme: Effect on protein structure and physical stability. Eur. J. Pharm. Biopharm. 2014, 88, 325–331. 10.1016/j.ejpb.2014.09.001. 25218319

[ref24] Kayitmazer A. B.; Seeman D.; Minsky B. B.; Dubin P. L.; Xu Y. Protein–polyelectrolyte interactions. Soft Matter 2013, 9, 2553–2583. 10.1039/c2sm27002a.

[ref25] Hyman A. A.; Weber C. A.; Jülicher F. Liquid-Liquid Phase Separation in Biology. Annu. Rev. Cell Dev. Biol. 2014, 30, 39–58. 10.1146/annurev-cellbio-100913-013325. 25288112

[ref26] Lin Y.; McCarty J.; Rauch J. N.; Delaney K. T.; Kosik K. S.; Fredrickson G. H.; Shea J.-E.; Han S. Narrow equilibrium window for complex coacervation of tau and RNA under cellular conditions. eLife 2019, 8, e42571 10.7554/eLife.42571. 30950394 PMC6450672

[ref27] Aumiller W. M.; Keating C. D. Phosphorylation-mediated RNA/peptide complex coacervation as a model for intracellular liquid organelles. Nat. Chem. 2016, 8, 129–137. 10.1038/nchem.2414. 26791895

[ref28] Zhu J.; Jiang L. Liquid–Liquid Phase Separation Bridges Physics, Chemistry, and Biology. Langmuir 2022, 38, 9043–9049. 10.1021/acs.langmuir.2c01358. 35856491

[ref29] Jacobs M. I.; Jira E. R.; Schroeder C. M. Understanding How Coacervates Drive Reversible Small Molecule Reactions to Promote Molecular Complexity. Langmuir 2021, 37, 14323–14335. 10.1021/acs.langmuir.1c02231. 34856104

[ref30] McCall P. M.; Srivastava S.; Perry S. L.; Kovar D. R.; Gardel M. L.; Tirrell M. V. Partitioning and Enhanced Self-Assembly of Actin in Polypeptide Coacervates. Biophys. J. 2018, 114, 1636–1645. 10.1016/j.bpj.2018.02.020. 29642033 PMC5954293

[ref31] Martin N.; Li M.; Mann S. Selective Uptake and Refolding of Globular Proteins in Coacervate Microdroplets. Langmuir 2016, 32, 5881–5889. 10.1021/acs.langmuir.6b01271. 27268140

[ref32] Lindhoud S.; Claessens M. M. A. E. Accumulation of small protein molecules in a macroscopic complex coacervate. Soft Matter 2016, 12, 408–413. 10.1039/C5SM02386F. 26477852

[ref33] Blocher McTigue W. C.; Perry S. L. Protein Encapsulation Using Complex Coacervates: What Nature Has to Teach Us. Small 2020, 16, 1907671 10.1002/smll.201907671. 32363758

[ref34] Zhang Y.; Han K.; Lu D.; Liu Z. Reversible encapsulation of lysozyme within mPEG-b-PMAA: experimental observation and molecular dynamics simulation. Soft Matter 2013, 9, 8723–8729. 10.1039/c3sm50586c.

[ref35] Zhao M.; Zacharia N. S. Protein encapsulation via polyelectrolyte complex coacervation: Protection against protein denaturation. J. Chem. Phys. 2018, 149, 163326 10.1063/1.5040346. 30384671

[ref36] Xu Y.; Mazzawi M.; Chen K.; Sun L.; Dubin P. L. Protein Purification by Polyelectrolyte Coacervation: Influence of Protein Charge Anisotropy on Selectivity. Biomacromolecules 2011, 12, 1512–1522. 10.1021/bm101465y. 21413681

[ref37] Blocher McTigue W. C.; Perry S. L. Design rules for encapsulating proteins into complex coacervates. Soft Matter 2019, 15, 3089–3103. 10.1039/C9SM00372J. 30916112

[ref38] Kapelner R. A.; Obermeyer A. C. Ionic polypeptide tags for protein phase separation. Chem. Sci. 2019, 10, 2700–2707. 10.1039/C8SC04253E. 30996987 PMC6419950

[ref39] Obermeyer A. C.; Mills C. E.; Dong X.-H.; Flores R. J.; Olsen B. D. Complex coacervation of supercharged proteins with polyelectrolytes. Soft Matter 2016, 12, 3570–3581. 10.1039/C6SM00002A. 26965053

[ref40] van Lente J.; Pazos Urrea M.; Brouwer T.; Schuur B.; Lindhoud S. Complex coacervates as extraction media. Green Chem. 2021, 23, 5812–5824. 10.1039/D1GC01880A. 34456626 PMC8366913

[ref41] Black K. A.; Priftis D.; Perry S. L.; Yip J.; Byun W. Y.; Tirrell M. Protein Encapsulation via Polypeptide Complex Coacervation. ACS Macro Lett. 2014, 3, 1088–1091. 10.1021/mz500529v. 35610798

[ref42] van Lente J. J.; Claessens M. M. A. E.; Lindhoud S. Charge-Based Separation of Proteins Using Polyelectrolyte Complexes as Models for Membraneless Organelles. Biomacromolecules 2019, 20, 3696–3703. 10.1021/acs.biomac.9b00701. 31418555 PMC6794638

[ref43] Spruijt E.; Westphal A. H.; Borst J. W.; Cohen Stuart M. A.; van der Gucht J. Binodal Compositions of Polyelectrolyte Complexes. Macromolecules 2010, 43, 6476–6484. 10.1021/ma101031t.

[ref44] Chollakup R.; Smitthipong W.; Eisenbach C. D.; Tirrell M. Phase Behavior and Coacervation of Aqueous Poly(acrylic acid)-Poly(allylamine) Solutions. Macromolecules 2010, 43, 2518–2528. 10.1021/ma902144k.

[ref45] Chollakup R.; Beck J. B.; Dirnberger K.; Tirrell M.; Eisenbach C. D. Polyelectrolyte Molecular Weight and Salt Effects on the Phase Behavior and Coacervation of Aqueous Solutions of Poly(acrylic acid) Sodium Salt and Poly(allylamine) Hydrochloride. Macromolecules 2013, 46, 2376–2390. 10.1021/ma202172q.

[ref46] Priftis D.; Tirrell M. Phase behaviour and complex coacervation of aqueous polypeptide solutions. Soft Matter 2012, 8, 9396–9405. 10.1039/C2SM25604E.

[ref47] Li L.; Srivastava S.; Meng S.; Ting J. M.; Tirrell M. V. Effects of Non-Electrostatic Intermolecular Interactions on the Phase Behavior of pH-Sensitive Polyelectrolyte Complexes. Macromolecules 2020, 53, 7835–7844. 10.1021/acs.macromol.0c00999.

[ref48] Schröder P.; Cord-Landwehr S.; Schönhoff M.; Cramer C. Composition and Charge Compensation in Chitosan/Gum Arabic Complex Coacervates in Dependence on pH and Salt Concentration. Biomacromolecules 2023, 24, 1194–1208. 10.1021/acs.biomac.2c01255. 36779888

[ref49] Chang L.-W.; Lytle T. K.; Radhakrishna M.; Madinya J. J.; Vélez J.; Sing C. E.; Perry S. L. Sequence and entropy-based control of complex coacervates. Nat. Commun. 2017, 8, 1273 10.1038/s41467-017-01249-1. 29097695 PMC5668414

[ref50] Priftis D.; Laugel N.; Tirrell M. Thermodynamic Characterization of Polypeptide Complex Coacervation. Langmuir 2012, 28, 15947–15957. 10.1021/la302729r. 23083137

[ref51] Fu J.; Schlenoff J. B. Driving Forces for Oppositely Charged Polyion Association in Aqueous Solutions: Enthalpic, Entropic, but Not Electrostatic. J. Am. Chem. Soc. 2016, 138, 980–990. 10.1021/jacs.5b11878. 26771205

[ref52] Wang Q.; Schlenoff J. B. The Polyelectrolyte Complex/Coacervate Continuum. Macromolecules 2014, 47, 3108–3116. 10.1021/ma500500q.

[ref53] Meng S.; Ting J. M.; Wu H.; Tirrell M. V. Solid-to-Liquid Phase Transition in Polyelectrolyte Complexes. Macromolecules 2020, 53, 7944–7953. 10.1021/acs.macromol.0c00930.

[ref54] Liu Y.; Momani B.; Winter H. H.; Perry S. L. Rheological characterization of liquid-to-solid transitions in bulk polyelectrolyte complexes. Soft Matter 2017, 13, 7332–7340. 10.1039/C7SM01285C. 28951897

[ref55] Chen Y.; Yang M.; Shaheen S. A.; Schlenoff J. B. Influence of Nonstoichiometry on the Viscoelastic Properties of a Polyelectrolyte Complex. Macromolecules 2021, 54, 7890–7899. 10.1021/acs.macromol.1c01154.

[ref56] Tirrell M. Polyelectrolyte Complexes: Fluid or Solid?. ACS Cent. Sci. 2018, 4, 532–533. 10.1021/acscentsci.8b00284. 29805998 PMC5968439

[ref57] Huang J.; Morin F. J.; Laaser J. E. Charge-Density-Dominated Phase Behavior and Viscoelasticity of Polyelectrolyte Complex Coacervates. Macromolecules 2019, 52, 4957–4967. 10.1021/acs.macromol.9b00036.

[ref58] Qin J.; Priftis D.; Farina R.; Perry S. L.; Leon L.; Whitmer J.; Hoffmann K.; Tirrell M.; de Pablo J. J. Interfacial Tension of Polyelectrolyte Complex Coacervate Phases. ACS Macro Lett. 2014, 3, 565–568. 10.1021/mz500190w. 35590728

[ref59] Audus D. J.; Ali S.; Rumyantsev A. M.; Ma Y.; de Pablo J. J.; Prabhu V. M. Molecular Mass Dependence of Interfacial Tension in Complex Coacervation. Phys. Rev. Lett. 2021, 126, 237801 10.1103/PhysRevLett.126.237801. 34170179 PMC10168025

[ref60] Sing C. E. Development of the modern theory of polymeric complex coacervation. Adv. Colloid Interface Sci. 2017, 239, 2–16. 10.1016/j.cis.2016.04.004. 27161661

[ref61] Rubinstein M.; Liao Q.; Panyukov S. Structure of Liquid Coacervates Formed by Oppositely Charged Polyelectrolytes. Macromolecules 2018, 51, 9572–9588. 10.1021/acs.macromol.8b02059. 30853717 PMC6402498

[ref62] Rumyantsev A. M.; Zhulina E. B.; Borisov O. V. Complex Coacervate of Weakly Charged Polyelectrolytes: Diagram of States. Macromolecules 2018, 51, 3788–3801. 10.1021/acs.macromol.8b00342.

[ref63] Castelnovo M.; Joanny J.-F. Eur. Phys. J. E: Soft Matter Biol. Phys. 2001, 6, 377–386. 10.1007/s10189-001-8051-7.

[ref64] Salehi A.; Larson R. G. A Molecular Thermodynamic Model of Complexation in Mixtures of Oppositely Charged Polyelectrolytes with Explicit Account of Charge Association/Dissociation. Macromolecules 2016, 49, 9706–9719. 10.1021/acs.macromol.6b01464.

[ref65] Kumari S.; Dwivedi S.; Podgornik R. On the nature of screening in Voorn–Overbeek type theories. J. Chem. Phys. 2022, 156, 244901 10.1063/5.0091721. 35778110

[ref66] Zhang P.; Alsaifi N. M.; Wu J.; Wang Z.-G. Polyelectrolyte complex coacervation: Effects of concentration asymmetry. J. Chem. Phys. 2018, 149, 163303 10.1063/1.5028524. 30384721

[ref67] Zhang P.; Shen K.; Alsaifi N. M.; Wang Z.-G. Salt Partitioning in Complex Coacervation of Symmetric Polyelectrolytes. Macromolecules 2018, 51, 5586–5593. 10.1021/acs.macromol.8b00726.

[ref68] Adhikari S.; Leaf M. A.; Muthukumar M. Polyelectrolyte complex coacervation by electrostatic dipolar interactions. J. Chem. Phys. 2018, 149, 163308 10.1063/1.5029268. 30384692

[ref69] Zhang P.; Wang Z.-G. Interfacial Structure and Tension of Polyelectrolyte Complex Coacervates. Macromolecules 2021, 54, 10994–11007. 10.1021/acs.macromol.1c01809.

[ref70] Sayko R.; Tian Y.; Liang H.; Dobrynin A. V. Charged Polymers: From Polyelectrolyte Solutions to Polyelectrolyte Complexes. Macromolecules 2021, 54, 7183–7192. 10.1021/acs.macromol.1c01171.

[ref71] Delaney K. T.; Fredrickson G. H. Theory of polyelectrolyte complexation—Complex coacervates are self-coacervates. J. Chem. Phys. 2017, 146, 224902 10.1063/1.4985568. 29166038

[ref72] Lytle T. K.; Sing C. E. Transfer matrix theory of polymer complex coacervation. Soft Matter 2017, 13, 7001–7012. 10.1039/C7SM01080J. 28840212

[ref73] Lytle T. K.; Sing C. E. Tuning chain interaction entropy in complex coacervation using polymer stiffness, architecture, and salt valency. Mol. Syst. Des. Eng. 2018, 3, 183–196. 10.1039/C7ME00108H.

[ref74] Lytle T. K.; Chang L.-W.; Markiewicz N.; Perry S. L.; Sing C. E. Designing Electrostatic Interactions via Polyelectrolyte Monomer Sequence. ACS Cent. Sci. 2019, 5, 709–718. 10.1021/acscentsci.9b00087. 31041391 PMC6487445

[ref75] Qin J.; de Pablo J. J. Criticality and Connectivity in Macromolecular Charge Complexation. Macromolecules 2016, 49, 8789–8800. 10.1021/acs.macromol.6b02113.

[ref76] Zhang R.; Shklovskii B. Phase diagram of solution of oppositely charged polyelectrolytes. Phys. A 2005, 352, 216–238. 10.1016/j.physa.2004.12.037.

[ref77] Perry S. L.; Sing C. E. PRISM-Based Theory of Complex Coacervation: Excluded Volume versus Chain Correlation. Macromolecules 2015, 48, 5040–5053. 10.1021/acs.macromol.5b01027.

[ref78] Radhakrishna M.; Basu K.; Liu Y.; Shamsi R.; Perry S. L.; Sing C. E. Molecular Connectivity and Correlation Effects on Polymer Coacervation. Macromolecules 2017, 50, 3030–3037. 10.1021/acs.macromol.6b02582.

[ref79] Yang M.; Sonawane S. L.; Digby Z. A.; Park J. G.; Schlenoff J. B. Influence of “Hydrophobicity” on the Composition and Dynamics of Polyelectrolyte Complex Coacervates. Macromolecules 2022, 55, 7594–7604. 10.1021/acs.macromol.2c00267.

[ref80] Kim S.; Lee M.; Lee W. B.; Choi S.-H. Ionic-Group Dependence of Polyelectrolyte Coacervate Phase Behavior. Macromolecules 2021, 54, 7572–7581. 10.1021/acs.macromol.1c00216.

[ref81] Fu J.; Fares H. M.; Schlenoff J. B. Ion-Pairing Strength in Polyelectrolyte Complexes. Macromolecules 2017, 50, 1066–1074. 10.1021/acs.macromol.6b02445.

[ref82] Li L.; Rumyantsev A. M.; Srivastava S.; Meng S.; de Pablo J. J.; Tirrell M. V. Effect of Solvent Quality on the Phase Behavior of Polyelectrolyte Complexes. Macromolecules 2021, 54, 105–114. 10.1021/acs.macromol.0c01000.

[ref83] Perry S. L.; Li Y.; Priftis D.; Leon L.; Tirrell M. The Effect of Salt on the Complex Coacervation of Vinyl Polyelectrolytes. Polymers 2014, 6, 1756–1772. 10.3390/polym6061756.

[ref84] Schlenoff J. B.; Yang M.; Digby Z. A.; Wang Q. Ion Content of Polyelectrolyte Complex Coacervates and the Donnan Equilibrium. Macromolecules 2019, 52, 9149–9159. 10.1021/acs.macromol.9b01755.

[ref85] Meng S.; Liu Y.; Yeo J.; Ting J. M.; Tirrell M. V. Effect of mixed solvents on polyelectrolyte complexes with salt. Colloid Polym. Sci. 2020, 298, 887–894. 10.1007/s00396-020-04637-0.

[ref86] Li L.; Srivastava S.; Andreev M.; Marciel A. B.; de Pablo J. J.; Tirrell M. V. Phase Behavior and Salt Partitioning in Polyelectrolyte Complex Coacervates. Macromolecules 2018, 51, 2988–2995. 10.1021/acs.macromol.8b00238.

[ref87] Dautzenberg H.; Kriz J. Response of Polyelectrolyte Complexes to Subsequent Addition of Salts with Different Cations. Langmuir 2003, 19, 5204–5211. 10.1021/la0209482.

[ref88] Iyer D.; Syed V. M. S.; Srivastava S. Influence of divalent ions on composition and viscoelasticity of polyelectrolyte complexes. J. Polym. Sci. 2021, 59, 2895–2904. 10.1002/pol.20210668.

[ref89] Huang S.; Zhao M.; Dawadi M. B.; Cai Y.; Lapitsky Y.; Modarelli D. A.; Zacharia N. S. Effect of small molecules on the phase behavior and coacervation of aqueous solutions of poly(diallyldimethylammonium chloride) and poly(sodium 4-styrene sulfonate). J. Colloid Interface Sci. 2018, 518, 216–224. 10.1016/j.jcis.2018.02.029. 29459301

[ref90] van Lente J. J.; Lindhoud S. Extraction of Lysozyme from Chicken Albumen Using Polyelectrolyte Complexes. Small 2022, 18, 2105147 10.1002/smll.202105147. 34877780

[ref91] Choi S.; Knoerdel A. R.; Sing C. E.; Keating C. D. Effect of Polypeptide Complex Coacervate Microenvironment on Protonation of a Guest Molecule. J. Phys. Chem. B 2023, 127, 5978–5991. 10.1021/acs.jpcb.3c02098. 37350455

[ref92] Landsgesell J.; Nova L.; Rud O.; Uhlik F.; Sean D.; Hebbeker P.; Holm C.; Košovan P. Simulations of ionization equilibria in weak polyelectrolyte solutions and gels. Soft Matter 2019, 15, 1155–1185. 10.1039/C8SM02085J. 30706070

[ref93] Gartner T. E. I.; Jayaraman A. Modeling and Simulations of Polymers: A Roadmap. Macromolecules 2019, 52, 755–786. 10.1021/acs.macromol.8b01836.

[ref94] Ou Z.; Muthukumar M. Entropy and enthalpy of polyelectrolyte complexation: Langevin dynamics simulations. J. Chem. Phys. 2006, 124, 154902 10.1063/1.2178803. 16674260

[ref95] Singh A. N.; Yethiraj A. Driving Force for the Complexation of Charged Polypeptides. J. Phys. Chem. B 2020, 124, 1285–1292. 10.1021/acs.jpcb.9b09553. 31990555

[ref96] Rathee V. S.; Sidky H.; Sikora B. J.; Whitmer J. K. Role of Associative Charging in the Entropy–Energy Balance of Polyelectrolyte Complexes. J. Am. Chem. Soc. 2018, 140, 15319–15328. 10.1021/jacs.8b08649. 30351015

[ref97] Chen S.; Zhang P.; Wang Z.-G. Complexation between Oppositely Charged Polyelectrolytes in Dilute Solution: Effects of Charge Asymmetry. Macromolecules 2022, 55, 3898–3909. 10.1021/acs.macromol.2c00339.

[ref98] Tsanai M.; Frederix P. J. M.; Schroer C. F. E.; Souza P. C. T.; Marrink S. J. Coacervate formation studied by explicit solvent coarse-grain molecular dynamics with the Martini model. Chem. Sci. 2021, 12, 8521–8530. 10.1039/D1SC00374G. 34221333 PMC8221187

[ref99] Shakya A.; Girard M.; King J. T.; Olvera de la Cruz M. Role of Chain Flexibility in Asymmetric Polyelectrolyte Complexation in Salt Solutions. Macromolecules 2020, 53, 1258–1269. 10.1021/acs.macromol.9b02355.

[ref100] Bobbili S. V.; Milner S. T. Closed-Loop Phase Behavior of Nonstoichiometric Coacervates in Coarse-Grained Simulations. Macromolecules 2022, 55, 511–516. 10.1021/acs.macromol.1c02115.

[ref101] Andreev M.; Prabhu V. M.; Douglas J. F.; Tirrell M.; de Pablo J. J. Complex Coacervation in Polyelectrolytes from a Coarse-Grained Model. Macromolecules 2018, 51, 6717–6723. 10.1021/acs.macromol.8b00556. PMC7722290 33299652

[ref102] Liang H.; de Pablo J. J. A Coarse-Grained Molecular Dynamics Study of Strongly Charged Polyelectrolyte Coacervates: Interfacial, Structural, and Dynamical Properties. Macromolecules 2022, 55, 4146–4158. 10.1021/acs.macromol.2c00246.

[ref103] Panagiotopoulos A. Z. Direct determination of phase coexistence properties of fluids by Monte Carlo simulation in a new ensemble. Mol. Phys. 1987, 61, 813–826. 10.1080/00268978700101491.

[ref104] Knoerdel A. R.; Blocher McTigue W. C.; Sing C. E. Transfer Matrix Model of pH Effects in Polymeric Complex Coacervation. J. Phys. Chem. B 2021, 125, 8965–8980. 10.1021/acs.jpcb.1c03065. 34328340

[ref105] Zheng B.; Avni Y.; Andelman D.; Podgornik R. Phase Separation of Polyelectrolytes: The Effect of Charge Regulation. J. Phys. Chem. B 2021, 125, 7863–7870. 10.1021/acs.jpcb.1c01986. 34232047 PMC8389888

[ref106] Landsgesell J.; Hebbeker P.; Rud O.; Lunkad R.; Košovan P.; Holm C. Grand-Reaction Method for Simulations of Ionization Equilibria Coupled to Ion Partitioning. Macromolecules 2020, 53, 3007–3020. 10.1021/acs.macromol.0c00260.

[ref107] Beyer D.; Holm C. A generalized grand-reaction method for modeling the exchange of weak (polyprotic) acids between a solution and a weak polyelectrolyte phase. J. Chem. Phys. 2023, 159, 014905 10.1063/5.0155973. 37417757

[ref108] Rahbari A.; Hens R.; Ramdin M.; Moultos O. A.; Dubbeldam D.; Vlugt T. J. H. Recent advances in the continuous fractional component Monte Carlo methodology. Mol. Simul. 2021, 47, 804–823. 10.1080/08927022.2020.1828585.

[ref109] Poursaeidesfahani A.; Hens R.; Rahbari A.; Ramdin M.; Dubbeldam D.; Vlugt T. J. H. Efficient Application of Continuous Fractional Component Monte Carlo in the Reaction Ensemble. J. Chem. Theory Comput. 2017, 13, 4452–4466. 10.1021/acs.jctc.7b00092. 28737933 PMC5597954

[ref110] Beyer D.; Landsgesell J.; Hebbeker P.; Rud O.; Lunkad R.; Košovan P.; Holm C. Correction to ”Grand-Reaction Method for Simulations of Ionization Equilibria Coupled to Ion Partitioning. Macromolecules 2022, 55, 1088 10.1021/acs.macromol.1c02672.

[ref111] Landsgesell J.; Beyer D.; Hebbeker P.; Košovan P.; Holm C. The pH-Dependent Swelling of Weak Polyelectrolyte Hydrogels Modeled at Different Levels of Resolution. Macromolecules 2022, 55, 3176–3188. 10.1021/acs.macromol.1c02489.

[ref112] Staňo R.; Košovan P.; Tagliabue A.; Holm C. Electrostatically Cross-Linked Reversible Gels-Effects of pH and Ionic Strength. Macromolecules 2021, 54, 4769–4781. 10.1021/acs.macromol.1c00470.

[ref113] Weeks J. D.; Chandler D.; Andersen H. C. Role of Repulsive Forces in Determining the Equilibrium Structure of Simple Liquids. J. Chem. Phys. 1971, 54, 5237–5247. 10.1063/1.1674820.

[ref114] Hansen J.; McDonald I. Theory of Simple Liquids: with Applications to Soft Matter; Elsevier Science, 2013.

[ref115] McQuarrie D. Statistical Mechanics; Chemistry Series; Harper & Row, 1975.

[ref116] Frenkel D.; Smit B. Understanding Molecular Simulation: From Algorithms to Applications; Computational Science Series; Academic Press, 2002.

[ref117] Hockney R. W.; Eastwood J. W. Computer Simulation Using Particles; IOP: London, 1988.

[ref118] Deserno M.; Holm C. How to mesh up Ewald sums. I. A theoretical and numerical comparison of various particle mesh routines. J. Chem. Phys. 1998, 109, 7678–7693. 10.1063/1.477414.

[ref119] Deserno M.; Holm C. How to mesh up Ewald sums. II. An accurate error estimate for the Particle-Particle-Particle-Mesh algorithm. J. Chem. Phys. 1998, 109, 7694–7701. 10.1063/1.477415.

[ref120] Digby Z. A.; Yang M.; Lteif S.; Schlenoff J. B. Salt Resistance as a Measure of the Strength of Polyelectrolyte Complexation. Macromolecules 2022, 55, 978–988. 10.1021/acs.macromol.1c02151.

[ref121] Beyer D.; Košovan P.; Holm C. Simulations explain the Swelling Behavior of Hydrogels with Alternating Neutral and Weakly Acidic Blocks. Macromolecules 2022, 55, 10751–10760. 10.1021/acs.macromol.2c01916.

[ref122] Lunkad R.; Murmiliuk A.; Hebbeker P.; Boublík M.; Tošner Z.; Štěpánek M.; Košovan P. Quantitative prediction of charge regulation in oligopeptides. Mol. Syst. Des. Eng. 2021, 6, 122–131. 10.1039/D0ME00147C.

[ref123] Lunkad R.; Murmiliuk A.; Tošner Z.; Štěpánek M.; Košovan P. Role of pKA in Charge Regulation and Conformation of Various Peptide Sequences. Polymers 2021, 13, 214 10.3390/polym13020214. 33435335 PMC7827592

[ref124] Weik F.; Weeber R.; Szuttor K.; Breitsprecher K.; de Graaf J.; Kuron M.; Landsgesell J.; Menke H.; Sean D.; Holm C. ESPResSo 4.0 – an extensible software package for simulating soft matter systems. Eur. Phys. J.: Spec. Top. 2019, 227, 1789–1816. 10.1140/epjst/e2019-800186-9.

[ref125] Lide D. R. CRC Handbook of Chemistry and Physics: A Ready-Reference Book of Chemical and Physical Data; CRC Press, 1995.

[ref126] Košovan P.; Richter T.; Holm C. Modeling of Polyelectrolyte Gels in Equilibrium with Salt Solutions. Macromolecules 2015, 48, 7698–7708. 10.1021/acs.macromol.5b01428.

[ref127] Smiatek J. Theoretical and Computational Insight into Solvent and Specific Ion Effects for Polyelectrolytes: The Importance of Local Molecular Interactions. Molecules 2020, 25, 1661 10.3390/molecules25071661. 32260301 PMC7180813

[ref128] Salis A.; Ninham B. W. Models and mechanisms of Hofmeister effects in electrolyte solutions, and colloid and protein systems revisited. Chem. Soc. Rev. 2014, 43, 7358–7377. 10.1039/C4CS00144C. 25099516

[ref129] An Y.; Singh S.; Bejagam K. K.; Deshmukh S. A. Development of an Accurate Coarse-Grained Model of Poly(acrylic acid) in Explicit Solvents. Macromolecules 2019, 52, 4875–4887. 10.1021/acs.macromol.9b00615.

[ref130] Giussi J. M.; Martínez Moro M.; Iborra A.; Cortez M. L.; Di Silvio D.; Llarena Conde I.; Longo G. S.; Azzaroni O.; Moya S. A study of the complex interaction between poly allylamine hydrochloride and negatively charged poly(N-isopropylacrylamide-co-methacrylic acid) microgels. Soft Matter 2020, 16, 881–890. 10.1039/C9SM02070E. 31942906

[ref131] Truesdell A. H. Activity Coefficients of Aqueous Sodium Chloride from 15° to 50°C Measured with a Glass Electrode. Science 1968, 161, 884–886. 10.1126/science.161.3844.884. 17812114

[ref132] Neitzel A. E.; Fang Y. N.; Yu B.; Rumyantsev A. M.; de Pablo J. J.; Tirrell M. V. Polyelectrolyte Complex Coacervation across a Broad Range of Charge Densities. Macromolecules 2021, 54, 6878–6890. 10.1021/acs.macromol.1c00703. 34334816 PMC8320234

[ref133] Duan X.; Nakamura I. A new lattice Monte Carlo simulation for dielectric saturation in ion-containing liquids. Soft Matter 2015, 11, 3566–3571. 10.1039/C5SM00336A. 25807274

[ref134] Jouyban A.; Soltanpour S.; Chan H.-K. A simple relationship between dielectric constant of mixed solvents with solvent composition and temperature. Int. J. Pharm. 2004, 269, 353–360. 10.1016/j.ijpharm.2003.09.010. 14706247

[ref135] Hebbeker P.; Blanco P.; Uhlík F.; Kosovan P. Finite-Size Effects in Simulations of Chemical Reactions. 2023, 10.26434/chemrxiv-2023-n2g58.

