# Supplementary material for: Sequestration of Small Ions and Weak Acids and Bases by a Polyelectrolyte Complex Studied by Simulation and Experiment
Source: Macromolecules. 2024 Jan 18;57(3):1383–98. doi: 10.1021/acs.macromol.3c01209 (PMC10867894; doi:10.1021/acs.macromol.3c01209)
Supplement: Supplementary file 1 — ma3c01209_si_001.pdf [file ma3c01209_si_001.pdf]

Supporting Information: Sequestration of small ions, weak acids and bases by polyelectrolyte complex studied by simulation and experiment

Roman Staňo,[*,†,‡] Jéré van Lente,[¶] Saskia Lindhoud,[¶] and Peter Košovan[§]

†*Faculty of Physics, University of Vienna, Boltzmanngasse 5, 1090 Vienna, Austria*

‡*Vienna Doctoral School in Physics, University of Vienna, Boltzmanngasse 5, 1090 Vienna, Austria*

¶*Department of Molecules & Materials, University of Twente, Drienerlolaan 5, 7522 NB Enschede, The Netherlands*

§*Department of Physical and Macromolecular Chemistry, Faculty of Science, Charles University, Hlavova 8, 128 40 Prague 2, Czechia*

E-mail: roman.stano@univie.ac.at

S1 Additional details on the simulation model and method

S1.1 Simulation parameters and units

First, in Figure S1 we plot the non-bonding potential from Eq. 5 employed between the monomeric units of the polymers. We can estimate the corresponding second virial coeffi-

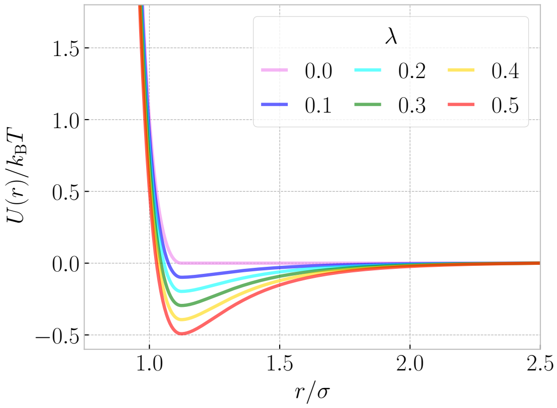

Figure S1: Non-bonded potential between monomers as a function of distance for different values of λ.

cients between monomers using the Mayer f-function[S1,S2] as

$$B_2 = -2\pi \int_0^\infty \mathrm{d}r\ r^2 \Big(\exp(-\beta U(r)) - 1 \Big), \tag{S1}$$

as listed in Tab. S1. We see that for free uncharged monomers, transition from repulsion-dominated two-body interactions ($B_2 > 0$) to attraction-dominated ($B_2 < 0$) occurs somewhere between $\lambda \in (0.4, 0.5)$, where the monomers reach their θ-state conditions. For the polymer composed of such monomers, there are several definitions of θ-state, differing in whether one considers B_2 for monomers, blobs or whole chains.[S2] Nevertheless, simulations of neutral polymers[S3] suggest that the θ-point stays close to $\lambda \sim 0.4$ and is rather insensitive to topology of the chain. Accordingly, our choice of λ parameters covers a range of conditions from athermal solvent to θ-state.

S1.2 Linear dependence between equilibrium constants

For the equilibrium constant from Eq. 9 we can write:

$$\Gamma_{(\mathrm{Na}^+,\mathrm{Cl}^-)} = \gamma_{\mathrm{Na}^+}[\mathrm{Na}^+]\ \gamma_{\mathrm{Cl}^-}[\mathrm{Cl}^-], \tag{S2}$$

Table S1: Mapping λ values from Eq. 5 onto the second virial coefficients between free monomers

λ	B_2 [σ^3]	B_2 [cm^3/mol]
0.0	2.205	59.4
0.1	1.725	46.5
0.2	1.218	32.8
0.3	0.680	18.3
0.4	0.110	2.9
0.5	−0.496	−13.4

Table S2: Excluded-volume length scale and charge number for all the types of the particles occuring in our systems.

	polymer		salt ions				additional solutes			
	PAA^-	PAH^+	Na^+	Cl^-	H^+	OH^-	M^{2+}	H_2SuA	$HSuA^-$	SuA^{2-}
σ_i/σ	1.20	1.20	1.00	1.00	1.00	1.00	1.00	1.00	1.00	1.00
z_i	−1	+1	+1	−1	+1	−1	+2	0	−1	−2

where $[i] = c_i/c_i^{\ominus}$ and γ_i is the activity coefficient. Analogically,

$$\Gamma_{(\mathrm{H^+,Cl^-})} = \gamma_{\mathrm{H^+}}[\mathrm{H^+}]\ \gamma_{\mathrm{Cl^-}}[\mathrm{Cl^-}], \tag{S3}$$

$$\Gamma_{(\mathrm{H^+,OH^-})} = \gamma_{\mathrm{H^+}}[\mathrm{H^+}]\ \gamma_{\mathrm{OH^-}}[\mathrm{OH^-}], \tag{S4}$$

$$\Gamma_{(\mathrm{Na^+,OH^-})} = \gamma_{\mathrm{Na^+}}[\mathrm{Na^+}]\ \gamma_{\mathrm{OH^-}}[\mathrm{OH^-}], \tag{S5}$$

where we see

$$\Gamma_{(\mathrm{Na^+,OH^-})} = \frac{\gamma_{\mathrm{Na^+}}[\mathrm{Na^+}]\ \gamma_{\mathrm{OH^-}}[\mathrm{OH^-}]\ \gamma_{\mathrm{H^+}}[\mathrm{H^+}]\ \gamma_{\mathrm{Cl^-}}[\mathrm{Cl^-}]}{\gamma_{\mathrm{H^+}}[\mathrm{H^+}]\ \gamma_{\mathrm{Cl^-}}[\mathrm{Cl^-}]} = \frac{\Gamma_{(\mathrm{Na^+,Cl^-})}\ \Gamma_{(\mathrm{H^+,OH^-})}}{\Gamma_{(\mathrm{H^+,Cl^-})}}, \tag{S6}$$

thereby choice of $\Gamma_{(\mathrm{Na^+,Cl^-})}$ and $\Gamma_{(\mathrm{H^+,Cl^-})}$ together with the constant ionic product of water, $\Gamma_{(\mathrm{H^+,OH^-})}$, uniquely defines $\Gamma_{(\mathrm{Na^+,OH^-})}$.

Similarly for the succinic acid, the choice of $\mathrm{p}K_{\mathrm{A},1}$ and $\mathrm{p}K_{\mathrm{A},2}$ together with the equilibrium constant, defined in Eq. 16, uniquely defines all other equilibrium constants, Eq. 17

and Eq. 18, describing the insertions of the other ionic forms of the succinic acid as follows

$$\Gamma_{(\mathrm{H_2SuA})} = \gamma_{\mathrm{H_2SuA}}[\mathrm{H_2SuA}], \tag{S7}$$

$$K_{\mathrm{A},1} = \frac{\gamma_{\mathrm{HSuA^-}}[\mathrm{HSuA^-}]\ \gamma_{\mathrm{H^+}}[\mathrm{H^+}]}{\gamma_{\mathrm{H_2SuA}}[\mathrm{H_2SuA}]}, \tag{S8}$$

$$K_{\mathrm{A},2} = \frac{\gamma_{\mathrm{SuA^{2-}}}[\mathrm{SuA^{2-}}]\ \gamma_{\mathrm{H^+}}[\mathrm{H^+}]}{\gamma_{\mathrm{HSuA^-}}[\mathrm{HSuA^-}]}, \tag{S9}$$

from where we have,

$$\Gamma_{(\mathrm{HSuA^-},\mathrm{H^+})} = \gamma_{\mathrm{HSuA^-}}[\mathrm{HSuA^-}]\ \gamma_{\mathrm{H^+}}[\mathrm{H^+}] = K_{\mathrm{A},1}\Gamma_{(\mathrm{H_2SuA})} \tag{S10}$$

$$\Gamma_{(\mathrm{SuA^{2-}},2\mathrm{H^+})} = \gamma_{\mathrm{SuA^{2-}}}[\mathrm{SuA^{2-}}]\ \gamma^2_{\mathrm{H^+}}[\mathrm{H^+}]^2 = K_{\mathrm{A},1}K_{\mathrm{A},2}\Gamma_{(\mathrm{H_2SuA})}. \tag{S11}$$

S1.3 Grand-reaction Monte Carlo setup

Following Sec. S1.2, it is possible to combine chemical equations listed in Sec. 2 to construct additional set of formal reactions, in order to enhance the Monte Carlo sampling of the compositions. The ionization reactions Eq. 14 and Eq. 15 can be formally added or subtracted from grandcanonical coupling reactions, Eq. 9, Eq. 10, Eq. 11 and Eq. 12, yielding composite ionization-insertion reactions Eq. S12, Eq. S13, Eq. S14, Eq. S15, Eq. S16, Eq. S17, whose equilibrium constants are uniquely defined.[S4]

$$\mathrm{H_2SuA} \rightleftharpoons \mathrm{HSuA^-} + \mathrm{Na^+}, \qquad K_{\mathrm{A},1}\Gamma_{(\mathrm{Na^+},\mathrm{Cl^-})}/\Gamma_{(\mathrm{H^+},\mathrm{Cl^-})}, \tag{S12}$$

$$\mathrm{HSuA^-} \rightleftharpoons \mathrm{SuA^{2-}} + \mathrm{Na^+}, \qquad K_{\mathrm{A},2}\Gamma_{(\mathrm{Na^+},\mathrm{Cl^-})}/\Gamma_{(\mathrm{H^+},\mathrm{Cl^-})}, \tag{S13}$$

$$\mathrm{H_2SuA} + \mathrm{OH^-} \rightleftharpoons \mathrm{HSuA^-}, \qquad K_{\mathrm{A},1}/\Gamma_{(\mathrm{H^+},\mathrm{OH^-})}, \tag{S14}$$

$$\mathrm{HSuA^-} + \mathrm{OH^-} \rightleftharpoons \mathrm{SuA^{2-}}, \qquad K_{\mathrm{A},2}/\Gamma_{(\mathrm{H^+},\mathrm{OH^-})}, \tag{S15}$$

$$\mathrm{H_2SuA} + \mathrm{Cl^-} \rightleftharpoons \mathrm{HSuA^-}, \qquad K_{\mathrm{A},1}/\Gamma_{(\mathrm{H^+},\mathrm{Cl^-})}, \tag{S16}$$

$$\mathrm{HSuA^-} + \mathrm{Cl^-} \rightleftharpoons \mathrm{SuA^{2-}}, \qquad K_{\mathrm{A},2}/\Gamma_{(\mathrm{H^+},\mathrm{Cl^-})}, \tag{S17}$$

Since a typical system contains more $Na^+$and Cl^-ions as compared to $H^+$and OH^-ions, the above reactions Eq. S12, Eq. S13, Eq. S16 and Eq. S17 yield significantly higher acceptance rates than the forms involving $H^+$and OH^-ions, thereby allowing more efficient exploration of the composition space.

A speed-up of the sampling can be also achieved employing reactions, which do not require insertion of particles into the dense system, but rather just exchange the identities of the ions

$$\mathrm{Cl^-} \rightleftharpoons \mathrm{OH^-}, \qquad \Gamma_{(\mathrm{H^+},\mathrm{OH^-})}/\Gamma_{(\mathrm{H^+},\mathrm{Cl^-})}, \tag{S18}$$

$$\mathrm{Na^+} \rightleftharpoons \mathrm{H^+}, \qquad \Gamma_{(\mathrm{H^+},\mathrm{Cl^-})}/\Gamma_{(\mathrm{Na^+},\mathrm{Cl^-})}, \tag{S19}$$

$$\mathrm{Cl^-} \rightleftharpoons \mathrm{HSuA^-}, \qquad K_{\mathrm{A},1}\Gamma_{(\mathrm{H_2SuA})}/\Gamma_{(\mathrm{H^+},\mathrm{Cl^-})}, \tag{S20}$$

$$\mathrm{OH^-} \rightleftharpoons \mathrm{HSuA^-}, \qquad K_{\mathrm{A},1}\Gamma_{(\mathrm{H_2SuA})}/\Gamma_{(\mathrm{H^+},\mathrm{OH^-})}, \tag{S21}$$

and finally reactions

$$2\ \mathrm{Cl^-} \rightleftharpoons \mathrm{SuA^{2-}}, \qquad K_{\mathrm{A},1}K_{\mathrm{A},2}/\Gamma_{(\mathrm{H_2SuA})}/\Gamma^2_{(\mathrm{H^+},\mathrm{Cl^-})}, \tag{S22}$$

$$2\ \mathrm{OH^-} \rightleftharpoons \mathrm{SuA^{2-}}, \qquad K_{\mathrm{A},1}K_{\mathrm{A},2}/\Gamma_{(\mathrm{H_2SuA})}/\Gamma^2_{(\mathrm{H^+},\mathrm{OH^-})}, \tag{S23}$$

$$2\ \mathrm{Na^+} \rightleftharpoons \mathrm{M^{2+}}, \qquad \Gamma_{(\mathrm{M^{2+}},2\mathrm{Cl^-})}/\Gamma^2_{(\mathrm{Na^+},\mathrm{Cl^-})}, \tag{S24}$$

$$2\ \mathrm{H^+} \rightleftharpoons \mathrm{M^{2+}}, \qquad \Gamma_{(\mathrm{M^{2+}},2\mathrm{Cl^-})}/\Gamma^2_{(\mathrm{H^+},\mathrm{Cl^-})}, \tag{S25}$$

completing the list of the used reactions.

S1.4 Measuring compositions in the low-concentration limit

Concentrations of the species exchanged with the reservoir can be accurately measured only if the box contains enough ions. In our experience, if the estimator of the mean of number of species of a given kind, is $\lesssim 10$, convergence of the quantity to its ensemble average is very slow. This poses a problem for simulations involving the exchange of succinic acid, whose

dominant form, without loss of generality, in the limit of high pH is SuA^{2-}. Accordingly, the mean counts of forms $HSuA^{-}$an H_2SuA are low and the concentrations can not be determined accurately due to the finite-size effects on the fluctuations.[S5] If this happens, we use the following alternative way to calculate the mean concentrations. Rearranging Eq. S7 shows that

$$[\mathrm{H_2SuA}] = \frac{\Gamma_{(\mathrm{H_2SuA})}}{\gamma_{\mathrm{H_2SuA}}} = \frac{\Gamma_{(\mathrm{H_2SuA})}}{\exp(\mu^{\mathrm{ex}}_{\mathrm{H_2SuA}})}, \tag{S26}$$

where the equilibrium constant is known, and we measure the excess chemical potential of H_2SuA using the Widom insertion,[S6] thus obtaining $[H_2SuA]$. The finite-size corrections to the Widom insertion[S7] can be readily applied and the convergence to the mean chemical potentials is faster than convergence of Grand-reaction insertions to the mean particle counts. Similarly, rearranging Eq. S12 and Eq. S12 for the other states of the acid, we obtain

$$[\mathrm{HSuA^-}] = \frac{K_{\mathrm{A},1}\Gamma_{\mathrm{Na^+,Cl^-}}}{\Gamma_{\mathrm{H^+,Cl^-}}} \frac{\exp(\mu^{\mathrm{ex}}_{\mathrm{H_2SuA}})}{\exp(\mu^{\mathrm{ex}}_{\mathrm{Na^+,HSuA^-}})} \frac{[\mathrm{H_2SuA}]}{[\mathrm{Na^+}]} \tag{S27}$$

$$[\mathrm{SuA^{2-}}] = \frac{K_{\mathrm{A},1}K_{\mathrm{A},2}\Gamma^2_{\mathrm{Na^+,Cl^-}}}{\Gamma^2_{\mathrm{H^+,Cl^-}}} \frac{\exp(\mu^{\mathrm{ex}}_{\mathrm{H_2SuA}})}{\exp(\mu^{\mathrm{ex}}_{\mathrm{2Na^+,SuA^{2-}}})} \frac{[\mathrm{H_2SuA}]}{[\mathrm{Na^+}]^2}, \tag{S28}$$

hence coupling the concentrations of the acid forms to the concentration of Na^+ ions, whose count in the box is always sufficiently high, so that the finite-size effects on the fluctuations are minuscule.

S2 Additional simulation results

S2.1 Structure factor

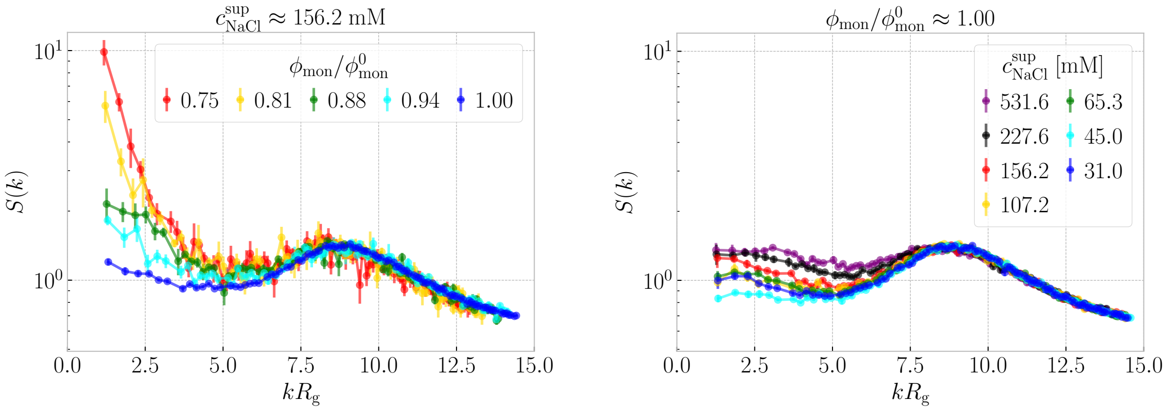

Figure S2: Static structure factor of monomer-monomer $(\mathrm{A}^- - \mathrm{A}^-)$ as a function of wavevector k, multiplied by radius of gyration of a single polyanion chain. The panel on the left shows development of the $k \to 0$ peak when moving to mean densities below the ϕ_{mon}^{0}. The panel on the right shows how $k \to 0$ plateau increases with increasing $c_{\mathrm{NaCl}}^{\mathrm{sup}}$.

S2.2 Dynamics of the system

The harmonic bonds in our model are soft enough to allow crossing of bonds, unlike the standard bead-spring model of Kremer and Grest.[S8] The bond crossing has little effect on the static structure and thermodynamic properties but it significantly affects the relaxation dynamics of polymer chains. The Kremer-Grest model was designed to study reptation dynamics of polymer chains in a melt which required that the crossing of bonds must be avoided. In contrast, our model is designed to study only thermodynamic and structural properties of the systems. Therefore, we allow bond crossing, which accelerates the relaxation dynamics, enabling faster equilibration and more efficient sampling of the simulated systems.

In Figure S3 we show the mean-square displacement of centers of mass of the polymer chains as a function of lag-time. We first observe the sub-diffusive regime with scaling exponent $\sim 1/2$ at short lag-times and eventually diffusive Brownian regime at large lag-times. Interestingly, these results agree very well with recent simulations of Liang *et al.*,[S9]

who used the polymer model derived from the Kremer-Grest model mentioned above. We surmise, that the bond crossing of our model is rather rare event and it does not destroy the equilibrium dynamics, but one would have to be extremely cautious using this model to measure properties such as viscosity, shear modulus or probing reptation and structure of entanglements in interpolyelectrolyte complexes out of equilibrium or at high densities.[S10]

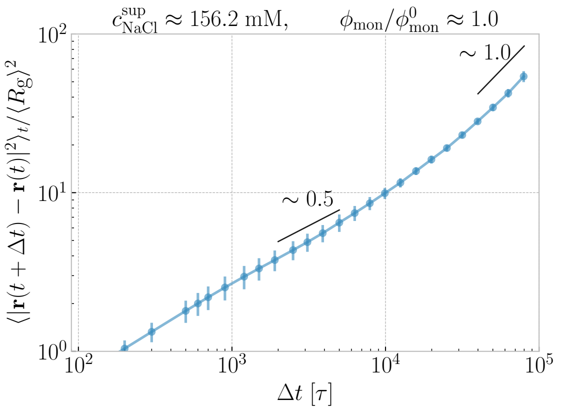

Figure S3: Mean squared displacement of the centers of mass of the chains as a function of lag time in a system in phase equilibrium with a selected c_{NaCl} sup. The displacement is averaged over all chains and all time origins and normalized by the mean radius of gyration squared.

S2.3 Effect of the size of the system

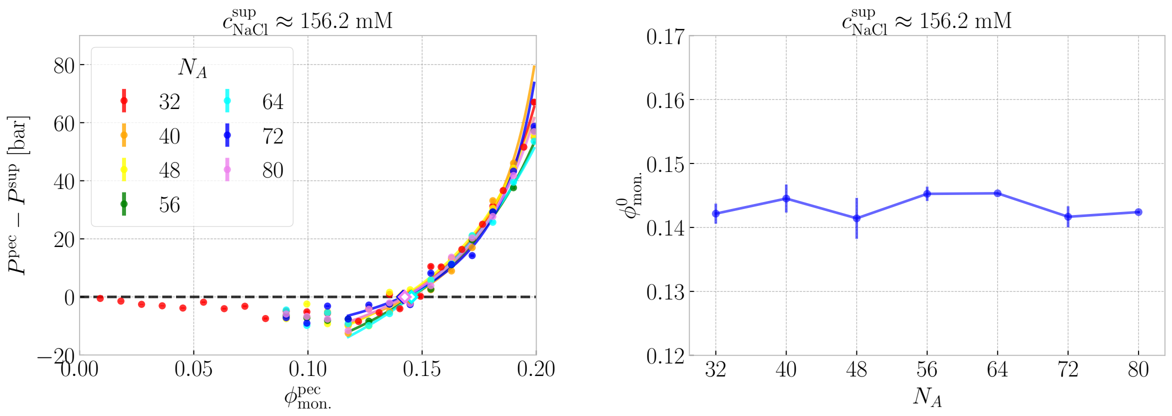

Figure S4: Pressure-composition protocol. Left panel: difference between the pressure of the interpolyelectrolyte complex phase and the NaCl supernatant as a function of monomer volume fraction in the interpolyelectrolyte complex phase. Circles are data from simulations for different system sizes (number of polyanion chains), lines are fits, hollow diamonds mark the intersection of the fits and the zero-pressure baseline, analogously to Figure 2. Right panel: the estimated equilibrium density (hollow diamonds from the left panel) shows only a weak dependence on the system size.

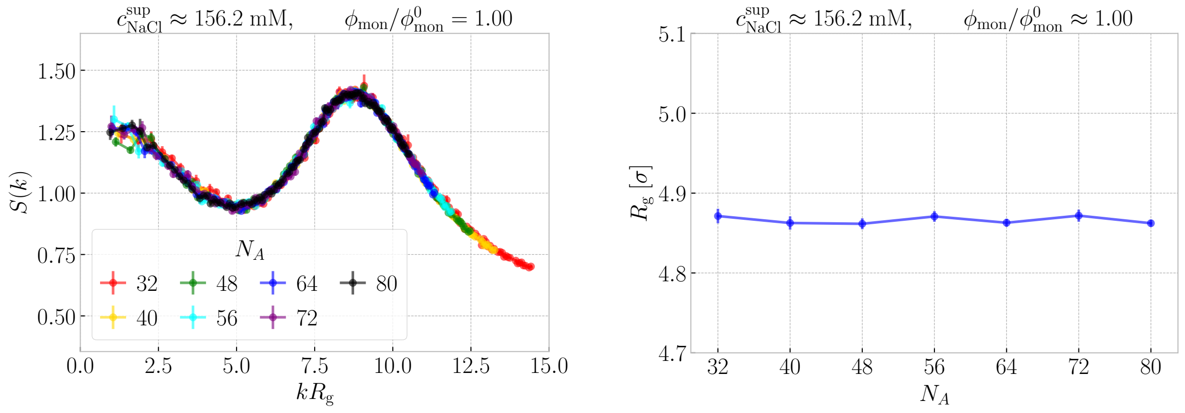

Figure S5: Structure of the system as a function of the size of the system. Left panel: Static structure factor of monomer-monomer ($\mathrm{A}^- - \mathrm{A}^-$) as a function of wavevector k, multiplied by radius of gyration of a single polyanion chain is nearly identical for many different system sizes (numbers of polyanion chains). Right panel: mean radius of gyration averaged over all chains as a function of number of chains.

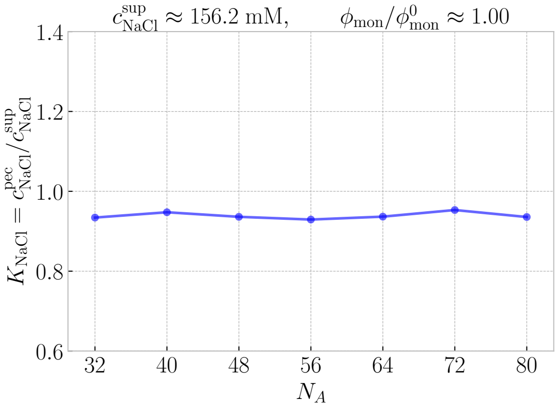

Figure S6: Partition coefficient of NaCl between the system and the supernatant of a selected c_{NaCl} sup, showing only weak dependence on the system size (number of polyanion chains).

S2.4 Debye-Hückel correction to the ideal charge response

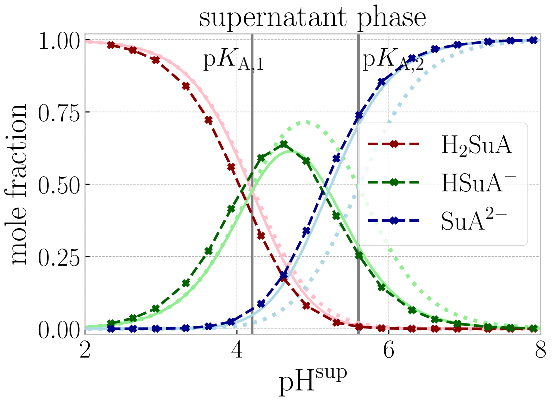

Figure S7: Mole fractions of ionization forms of the succinic acid in the supernatant phase as a function of pH of the supernatant. Dark symbols and lines are the simulation results, light dotted lines are mole fractions of an ideal system, light solid lines are ideal system with Debye-Hückel approximation for the activity coefficients.

Assuming that $\mathrm{pH} = -\log_{10}(\gamma_{\mathrm{H}^+}[\mathrm{H}^+])$, we can rearrange Eq. S8 and Eq. S9, which together with constraint $1 = x_{\mathrm{H_2SuA}} + x_{\mathrm{HSuA^-}} + x_{\mathrm{SuA^{2-}}}$ gives us mole fractions, x_i, of ionization forms

of the acid:

$$x_{\mathrm{H_2SuA}} = \frac{1}{1 + \dfrac{K_{\mathrm{A},1}\gamma_{\mathrm{H_2SuA}}}{\gamma_{\mathrm{HSuA^-}} \cdot 10^{-\mathrm{pH}}} + \dfrac{K_{\mathrm{A},1}K_{\mathrm{A},2}\gamma_{\mathrm{H_2SuA}}}{\gamma_{\mathrm{SuA^{2-}}} \cdot (10^{-\mathrm{pH}})^2}}, \tag{S29}$$

$$x_{\mathrm{HSuA^-}} = \frac{\dfrac{K_{\mathrm{A},1}\gamma_{\mathrm{H_2SuA}}}{\gamma_{\mathrm{HSuA^-}} \cdot 10^{-\mathrm{pH}}}}{1 + \dfrac{K_{\mathrm{A},1}\gamma_{\mathrm{H_2SuA}}}{\gamma_{\mathrm{HSuA^-}} \cdot 10^{-\mathrm{pH}}} + \dfrac{K_{\mathrm{A},1}K_{\mathrm{A},2}\gamma_{\mathrm{H_2SuA}}}{\gamma_{\mathrm{SuA^{2-}}} \cdot (10^{-\mathrm{pH}})^2}}, \tag{S30}$$

$$x_{\mathrm{SuA^{2-}}} = \frac{\dfrac{K_{\mathrm{A},1}K_{\mathrm{A},2}\gamma_{\mathrm{H_2SuA}}}{\gamma_{\mathrm{SuA^{2-}}} \cdot (10^{-\mathrm{pH}})^2}}{1 + \dfrac{K_{\mathrm{A},1}\gamma_{\mathrm{H_2SuA}}}{\gamma_{\mathrm{HSuA^-}} \cdot 10^{-\mathrm{pH}}} + \dfrac{K_{\mathrm{A},1}K_{\mathrm{A},2}\gamma_{\mathrm{H_2SuA}}}{\gamma_{\mathrm{SuA^{2-}}} \cdot (10^{-\mathrm{pH}})^2}}. \tag{S31}$$

In Figure S7 we plot the mole fractions as a function of pH, expanding on the Figure 5a) from the main text. In addition to the ideal mole fractions, where $\gamma = 1$ (light, dotted) and simulations (dark), we also present the ideal mole fractions with Debye-Hückel corrections for the activity of charge moieties, hence $\log_{10}(\gamma_i) \approx -Az_i^2\sqrt{I}$, where $A = 0.509\mathrm{mol}^{-1/2}\mathrm{L}^{1/2}$ and the constant ionic strength $I \approx 150\mathrm{mM}$, as it is dominated by the background NaCl salt. Overall, we can see that Debye-Hückel approximation is reasonable one within the range of explored parameters, as it is in a good agreement with the simulation results.

S3 Raw experimental data

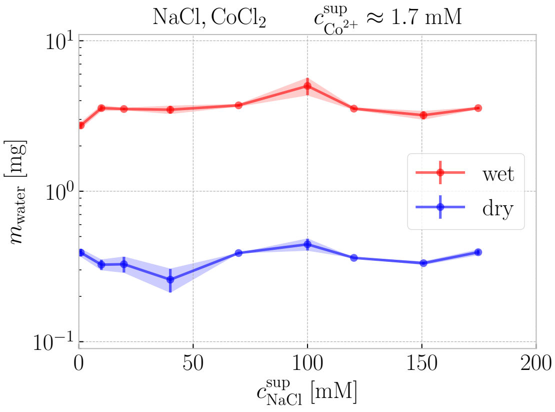

Figure S8: Mass of wet and dry PEC complexes with $CoCl_2$ as function of NaCl concentration in the supernatant.

We can determine the ratio of polymer concentrations in the two phases as

$$R = \frac{\frac{m^{pec}_{pol}}{V^{pec}}}{\frac{m^{sup}_{pol}}{V^{sup}}} \approx \frac{m^{sup} m^{pec}_{pol}}{m^{pec} m^{sup}_{pol}}, \tag{S32}$$

where the approximation is valid under assumption of equal mass densities of the two phases. Symbol $V^{(\cdot)}$ refers to the volume of the respective phase, m^{pec}_{pol} is the weight of the dry complex, m^{sup}_{pol} is calculated from the weight of the dry complex and the total polymer weight added into the system. Finally, m^{sup} is the weight of the supernatant and m^{pec} is the weight of wet complex.

References

(S1) McQuarrie, D. *Statistical Mechanics*; Chemistry Series; Harper & Row, 1975.

(S2) Rubinstein, M.; Colby, R. H. *Polymer Physics*; Oxford University Press, 2003.

(S3) Huissmann, S.; Blaak, R.; Likos, C. N. Star Polymers in Solvents of Varying Quality. *Macromolecules* **2009**, *42*, 2806–2816, DOI: 10.1021/ma8023359.

(S4) Landsgesell, J.; Hebbeker, P.; Rud, O.; Lunkad, R.; Košovan, P.; Holm, C. Grand-Reaction Method for Simulations of Ionization Equilibria Coupled to Ion Partitioning. *Macromolecules* **2020**, *53*, 3007–3020, DOI: 10.1021/acs.macromol.0c00260.

(S5) Hebbeker, P.; Blanco, P.; Uhlík, F.; Kosovan, P. Finite-Size Effects in Simulations of Chemical Reactions. 2023; https://doi.org/10.26434/chemrxiv-2023-n2g58, preprint.

(S6) Widom, B. Some Topics in the Theory of Fluids. *The Journal of Chemical Physics* **1963**, *39*, 2808–2812, DOI: 10.1063/1.1734110.

(S7) Siepmann, J. I.; McDonald, I. R.; Frenkel, D. Finite-size corrections to the chemical potential. *Journal of Physics: Condensed Matter* **1992**, *4*, 679, DOI: 10.1088/0953-8984/4/3/009.

(S8) Kremer, K.; Grest, G. S. Dynamics of entangled linear polymer melts: A molecular-dynamics simulation. *The Journal of Chemical Physics* **1990**, *92*, 5057–5086, DOI: 10.1063/1.458541.

(S9) Liang, H.; de Pablo, J. J. A Coarse-Grained Molecular Dynamics Study of Strongly Charged Polyelectrolyte Coacervates: Interfacial, Structural, and Dynamical Properties. *Macromolecules* **2022**, *55*, 4146–4158, DOI: 10.1021/acs.macromol.2c00246.

(S10) Yu, B.; Rauscher, P. M.; Jackson, N. E.; Rumyantsev, A. M.; de Pablo, J. J. Crossover from Rouse to Reptation Dynamics in Salt-Free Polyelectrolyte Complex Coacervates. *ACS Macro Letters* **2020**, *9*, 1318–1324, DOI: 10.1021/acsmacrolett.0c00522, PMID: 35638633.
